# Supplementary material for: Effects of s-ketamine and midazolam on respiratory variability: A randomized controlled pilot trial
Source: PLoS One. 2025 Sep 4;20(9):e0331358. doi: 10.1371/journal.pone.0331358 (PMC12410786; doi:10.1371/journal.pone.0331358)
Supplement: S1 Table — (DOCX) [file pone.0331358.s001.docx]

# Effects of s-ketamine and midazolam on respiratory variability: a randomized controlled pilot trial

# Supporting Information

**Table S1: Effects of S-ketamine and midazolam on respiratory parameters in patients with fibromyalgia.**

| ***Compared to saline condition*** | |  |
| --- | --- | --- |
|  | *Effect on mean respiratory rate (breaths/min)* |  |
| S-ketamine | - 0.619 (-1.596 to +0.394) |  |
| Midazolam | + 1.223 (-0.043 to 2.329) |  |
|  | *Effect on variability of respiratory rate* |  |
| S-ketamine | -0.014 (-0.058 to +0.034) |  |
| Midazolam | -0.071 (-0.121 to -0.019) | * |
|  | *Effect on variability of tidal volume* |  |
| S-ketamine | -0.062 (-0.119 to -0.003) | * |
| Midazolam | -0.117 (-0.170 to -0.062) | * |
| ***Compared to midazolam condition*** | | |
|  | *Effect on mean respiratory rate (breaths/min)* |  |
| S-ketamine | -1.834 (-2.773 to -0.789) | * |
| Saline | -1.223 (-2.329 to 0.043) |  |
|  | *Effect on variability of respiratory rate* |  |
| S-ketamine | 0.057 (0.012 to 0.101) | * |
| Saline | 0.071 (0.019 to 0.121) | * |
|  | *Effect on variability of tidal volume* |  |
| S-ketamine | 0.055 (0.002 to 0.109) | * |
| Saline | 0.117 (0.062 to 0.170) | * |

Patients with fibromyalgia were randomized to receive s-ketamine, midazolam, or saline in a blinded fashion, in three experiments. Changes in respiratory parameters were measured continuously using the thoracic bio-impedance method. Effect sizes are shown as Beta coefficient (95% confidence interval) in a linear mixed model with the difference in respiratory parameter before vs during study medication as the outcome variable, group allocation (s-ketamine, midazolam, or saline) as a fixed effect, and patient identifier as a random effect. Bootstrapping with 5,000 iterations was applied to generate empirical confidence intervals, reducing reliance on parametric assumptions and improving robustness in small-sample inference. Variability is calculated as the coefficient of variation, defined as the ratio between the standard deviation and the mean. * denotes statistical significance (p < 0.05).
