# Supplementary material for: Effects of s-ketamine and midazolam on respiratory variability: A randomized controlled pilot trial
Source: PLoS One. 2025 Sep 4;20(9):e0331358. doi: 10.1371/journal.pone.0331358 (PMC12410786; doi:10.1371/journal.pone.0331358)
Supplement: S2 File — (PDF) [file pone.0331358.s003.pdf]

RESEARCH PROTOCOL

The influence of pharmacological conditioning with S-ketamine on pain hypersensitivity in patients with fibromyalgia syndrome (FMS).

- May 2015: adaptation section 11.5: text in accordance to old and new Measure regarding Compulsory Insurance for Clinical Research in Humans
- Sept 2015: adaptation section 9.1, 9.2 and 12.5: text in accordance to WMO amendment on reporting SAE and temporary halt (section 10 of WMO)
- Oct 2015: adaptation section 4.4 – comment [CCMO15], 8.2 and 10.1 with respect to methodology/statistics
- Sept 2018: adaptation section 12.1 and comment [CCMO46] due to applicability GDPR as of May, 2018

PROTOCOL TITLE: The influence of pharmacological conditioning with S-ketamine on pain hypersensitivity in patients with fibromyalgia syndrome (FMS).

**Protocol ID**	**NL73444.058.21**
**Short title**	**Pharmacological conditioning with ketamine**
**EudraCT number**	2019-004812-73
**Version**	**6**
**Date**	8-6-2022
**Coordinating investigator/project leader**	Prof. dr. A.W.M. Evers<br>Full professor Health psychology, chair at the Health, Medical and Neuropsychology unit<br>Institute of Psychology<br>Faculty of Social and Behavioural Sciences<br>Leiden University<br>T: 071-527 3627, F 071-527 3619<br>E: a.evers@fsw.leidenuniv.nl
**Principal investigator Leiden University**<br><br><br><br><br><br><br><br>**Principal investigator VU University Medical Center**	Prof. dr. A.W.M. Evers<br>Full professor Health psychology, chair at the Health, Medical and Neuropsychology unit<br>Institute of Psychology<br>Faculty of Social and Behavioural Sciences<br>Leiden University<br>T: 071-527 3627, F 071-527 3619<br>E: a.evers@fsw.leidenuniv.nl<br><br>Prof. dr. M.A.H. Steegers, M.D.<br>Full professor in Anaesthesiology.<br>Specialization in Pain Medicine and Palliative Medicine.<br>Department of Anaesthesiology<br>VU University Medical Center<br>T: 020-444 1100<br>E: m.steegers@amsterdamumc.nl

**Sponsor (in Dutch: verrichter/opdrachtgever)**	Leiden University<br>Health, Medical and Neuropsychology unit<br>Institute of Psychology<br>Faculty of Social and Behavioural Sciences
**Subsidising party**	***Dutch Arthritis Society***
**Independent expert (s)**	Dr. C. Bulte, M.D.<br>Anesthesiologist<br>Department of Anesthesiology<br>Amsterdam UMC, location VUmc<br>T: 020-444 4383<br>E-mail: c.bulte@amsterdamumc.nl
**Medical responsible doctor**	Prof. dr. M.A.H. Steegers, M.D.<br>Full professor in Anaesthesiology.<br>Specialization in Pain Medicine and Palliative Medicine.<br>Department of Anesthesiology<br>Amsterdam UMC, location VUmc<br>T: 020-444 1100<br>E-mail: m.steegers@amsterdamumc.nl
**Laboratory sites**	Not applicable
**Pharmacy**	C.M. van der Hulst<br>Trial manager, Clinical Pharmacy and Toxicology<br>Leiden University Medical Center<br>Albinusdreef 2<br>2333 ZA Leiden<br>T: 071-526 4285<br>N. Bouwhuis

	GMP/GCP specialist, Clinical Pharmacy and Toxicology<br>Amsterdam University Medical Center<br>De Boelelaan 1117<br>1081 HV Amsterdam<br>T: 06 11450891

PROTOCOL SIGNATURE SHEET

Name	Signature	Date
**Sponsor or legal representative:**<br>Prof. dr. A.W.M. Evers<br>Full professor Health psychology, chair at Health, Medical and Neuropsychology unit<br>Institute of Psychology<br>Faculty of Social and Behavioural Sciences<br>Leiden University<br>T: 071-527 3627, F 071-527 3619<br>E: a.evers@fsw.leidenuniv.nl		9 June 2022
**Principal Investigators:**<br>Prof. dr. A.W.M. Evers<br>Full professor Health psychology, chair at Health, Medical and Neuropsychology unit<br>Institute of Psychology<br>Faculty of Social and Behavioural Sciences<br>Leiden University<br>T: 071-527 3627, F 071-527 3619<br>E: a.evers@fsw.leidenuniv.nl<br><br>Prof. dr. M.A.H. Steegers, M.D.<br>Full professor in Anaesthesiology.<br>Specialization in Pain Medicine and Palliative Medicine.<br>Department of Anaesthesiology<br>VU University Medical Center<br>T: 020-444 1100<br>E: m.steegers@amsterdamumc.nl		9 June 2022<br><br>09.06.22

TABLE OF CONTENTS

1. INTRODUCTION AND RATIONALE 12
2. OBJECTIVES 14
3. STUDY DESIGN 15
4. STUDY POPULATION 19
   - 4.1 Population (base) 19
   - 4.2 Inclusion criteria 19
   - 4.3 Exclusion criteria 19
   - 4.4 Sample size calculation 20
5. TREATMENT OF SUBJECTS 21
   - 5.1 Investigational product/treatment 21
   - 5.1 Use of co-intervention (if applicable) 22
   - 5.2 Escape medication (if applicable) 22
6. INVESTIGATIONAL PRODUCT 23
   - 6.1 Name and description of investigational product(s) 23
   - 6.2 Summary of findings from non-clinical studies 24
   - 6.3 Summary of findings from clinical studies 24
   - 6.4 Summary of known and potential risks and benefits 25
   - 6.5 Description and justification of route of administration and dosage 27
   - 6.6 Dosages, dosage modifications and method of administration 30
   - 6.7 Preparation and labelling of Investigational Medicinal Product 30
   - 6.8 Drug accountability 31
7. NON-INVESTIGATIONAL PRODUCT 31
   - 7.1 Name and description of non-investigational product(s) 31
   - 7.2 Summary of findings from non-clinical studies 31
   - 7.3 Summary of findings from clinical studies 31
   - 7.4 Summary of known and potential risks and benefits 31
   - 7.5 Description and justification of route of administration and dosage 32
   - 7.6 Dosages, dosage modifications and method of administration 32
   - 7.7 Preparation and labelling of Non Investigational Medicinal Product 32
   - 7.8 Drug accountability 32
8. METHODS 32
   - 8.1 Study parameters/endpoints 32
     - 8.1.1 Main study parameter/endpoint 32
     - 8.1.2 Secondary study parameters/endpoints (if applicable) 33
     - 8.1.3 Other study parameters (if applicable) 36
   - 8.2 Randomisation, blinding and treatment allocation 36
   - 8.3 Study procedures 37
   - 8.4 Withdrawal of individual subjects 43
     - 8.4.1 Specific criteria for withdrawal (if applicable) 44
   - 8.5 Replacement of individual subjects after withdrawal 44
   - 8.6 Follow-up of subjects withdrawn from treatment 44

8.7 Premature termination of the study 44
9. SAFETY REPORTING 44
9.1 Temporary halt for reasons of subject safety 44
9.2 AEs, SAEs and SUSARs 44
9.2.1 Adverse events (AEs) 44
9.2.2 Serious adverse events (SAEs) 45
9.2.3 Suspected unexpected serious adverse reactions (SUSARs) 45
9.3 Annual safety report 46
9.4 Follow-up of adverse events 47
9.5 [Data Safety Monitoring Board (DSMB) / Safety Committee] 47
10. STATISTICAL ANALYSIS 47
10.1 Primary study parameter(s) 47
10.2 Secondary study parameter(s) 48
10.3 Other study parameters 48
10.4 Interim analysis (if applicable) 49
11. ETHICAL CONSIDERATIONS 49
11.1 Regulation statement 49
11.2 Recruitment and consent 49
11.3 Benefits and risks assessment, group relatedness 49
11.4 Compensation for injury 51
11.5 Incentives (if applicable) 51
12. ADMINISTRATIVE ASPECTS, MONITORING AND PUBLICATION 51
12.1 Handling and storage of data and documents 51
12.2 Monitoring and Quality Assurance 51
12.3 Amendments 52
12.4 Annual progress report 52
12.5 Temporary halt and (prematurely) end of study report 52
12.6 Public disclosure and publication policy 53
13. STRUCTURED RISK ANALYSIS 53
13.1 Potential issues of concern 53
13.2 Synthesis 59
14. REFERENCES 60

LIST OF ABBREVIATIONS AND RELEVANT DEFINITIONS

**ABR**	**General Assessment and Registration form (ABR form), the application form that is required for submission to the accredited Ethics Committee; in Dutch: Algemeen Beoordelings- en Registratieformulier (ABR-formulier)**
**ACR**	**American College of Rheumatology**
**AE**	**Adverse Event**
**AR**	**Adverse Reaction**
**BMI**	**Body Mass Index**
**CA**	**Competent Authority**
**CCMO**	**Central Committee on Research Involving Human Subjects; in Dutch: Centrale Commissie Mensgebonden Onderzoek**
**CS**	**Central Sensitization**
**CV**	**Curriculum Vitae**
**DSMB**	**Data Safety Monitoring Board**
**EU**	**European Union**
**EudraCT**	**European drug regulatory affairs Clinical Trials**
**FIQ-R**	**Fibromyalgia Impact Questionnaire Revised**
**FMS**	**Fibromyalgia Syndrome**
**GABA**	**Gamma-amino-butiric-acid**
**GCP**	**Good Clinical Practice**
**GDPR**	**General Data Protection Regulation; in Dutch: Algemene Verordening Gegevensbescherming (AVG)**
**HADS**	**Hospital Anxiety and Depression Scale**
**IB**	**Investigator's Brochure**
**IC**	**Informed Consent**
**IMP**	**Investigational Medicinal Product**
**IMPD**	**Investigational Medicinal Product Dossier**
**METC**	**Medical research ethics committee (MREC); in Dutch: medisch-ethische toetsingscommissie (METC)**
**NMDA**	**N-methyl-D-aspartate**
**NRS**	**Numeric Rating Scale**
**NSAID**	**Non-Steroidal Anti Inflammatory Drug**
**NYHA**	**New York Heart Association**
**QST**	**Quantitative Sensory Testing**
**(S)AE**	**(Serious) Adverse Event**
**SE**	**Side effect**

**SPC**	**Summary of Product Characteristics; in Dutch: officiële productinformatie IB1-tekst**
**Sponsor**	**The sponsor is the party that commissions the organisation or performance of the research, for example a pharmaceutical company, academic hospital, scientific organisation or investigator. A party that provides funding for a study but does not commission it is not regarded as the sponsor, but referred to as a subsidising party.**
**SUSAR**	**Suspected Unexpected Serious Adverse Reaction**
**UAVG**	**Dutch Act on Implementation of the General Data Protection Regulation; in Dutch: Uitvoeringswet AVG**
**WMO**	**Medical Research Involving Human Subjects Act; in Dutch: Wet Medisch-wetenschappelijk Onderzoek met Mensen**

SUMMARY

Rationale: Patients with fibromyalgia syndrome (FMS) experience chronic widespread pain and an increased pain sensitivity. Although the exact etiology of FMS is unknown, there is prominent evidence supporting an important role of the CNS in the pain hypersensitivity, mainly due to central sensitization (CS). In CS, the central nervous system is in a state of hyperexcitability due to the involvement of glutamate at the NMDA receptor level. S(+)-ketamine (an NMDA receptor antagonist) has been proven an effective treatment for increased pain sensitivity in FMS, but potential serious side effects are a downside to its use. One solution could be the application of pharmacological conditioning. The learned somatic response of conditioning with S(+)-ketamine in patients might cause analgesia with a lower required dose. This has the potential to lower the chance of side effects seen in S(+)-ketamine administration. Pharmacological conditioning with S(+)-ketamine in FMS patients has not been investigated before.

Objective: The primary objective is to investigate whether pharmacological conditioning with S(+)-ketamine compared to pharmacological conditioning with placebo medication reduces pain hypersensitivity in patients with FMS.

Study design: This 3-arm randomized controlled trial consists of an S(+)-ketamine conditioning, active placebo (midazolam) conditioning and passive placebo (saline) conditioning group. Participants will go through an acquisition and an evocation phase. During the acquisition phase, participants will receive either S(+)-ketamine, midazolam or saline once a week for three weeks. In the evocation phase, all studied participants will receive a saline infusion. Outcome assessment takes place in the acquisition and evocation phase. Lower scores in pain sensitivity during evocation in the S(+)-ketamine group as compared to the placebo groups is considered evidence for pharmacological conditioning.

Study population: Dutch female FMS patients between 18 and 75 years of age without severe co-morbidities or contra-indications for investigational medicinal products are included in the study.

Intervention (if applicable): The S(+)-ketamine conditioning group receives a one-hour administration of intravenous (IV) S(+)-ketamine, with a step-up dose regimen (0.1 – 0.2 – 0.3mg/kg/h), once a week, for three weeks in the acquisition phase. The active placebo group receives a one-hour administration of IV midazolam, with a similar step-up dose regimen (0.017 – 0.033 – 0.05mg/kg/h), once a week, for three weeks. The passive placebo

group receives a one-hour administration of 50ml of IV saline, once a week, for three weeks. In the testing phase participants of all groups will receive 50 ml of IV saline, for one hour.

Main study parameters/endpoints: Pain sensitivity is assessed in this study by investigating three different Quantitative Sensory Testing (QST) modalities; pain pressure thresholds (PPT's), wind-up pain and aftersensations. The main endpoint for this study is the change in pain pressure threshold levels from baseline measured with pressure stimuli due to the pharmacological conditioning with S(+)-ketamine compared to pharmacological conditioning with placebo medication. PPT's are assessed at two different body locations; hand and lower leg. The secondary endpoints are: change in temporal summation or wind-up pain, aftersensations, clinical pain intensity, and self-reported disease activity due to pharmacological conditioning, differences in quantitative sensory testing (QST's) between body locations, extinction of pharmacological conditioning, variability of breathing and medication side effects.

Nature and extent of the burden and risks associated with participation, benefit and group relatedness: The study will last five weeks, with 4 hospital visits once a week. Every hospital visit will last 5 hours and includes; two hours screening-checks and baseline measurements, one-hour administration of medicine, and two-hours of post-intervention measurements. The doses of S(+)-ketamine and midazolam are low and associated with minimal expected side effects. QST is executed with a manual pressure pain algometer and implemented with standardized, well-validated and previously reported procedures. The algometer (Force Dial; Wagner Instruments, Greenwich, CT) has also been used in previous studies with patients with FMS [1, 2]. The respiratory volume monitor (ExSpiron, Respiratory Motion, Walthan, MA) uses an adhesive, non-invasive, thoracic electrode to measure respiratory rate and changes in tidal volume over time. Patients with FMS might benefit temporarily from the administration of S(+)-ketamine or placebo effects due to their pain-lowering effect. Also participants will receive a reimbursement of €120,- for completion of the study.

1. INTRODUCTION AND RATIONALE

Patients with FMS often experience chronic widespread pain (CWP) and pain hypersensitivity along with symptoms of depression, anxiety, fatigue and sleep disturbances [3, 4]. The exact origin of the increased sensitivity to pain in FMS, defined as nociplastic pain, is still unknown [5, 6]. However, evidence for pain hypersensitization in FMS currently points more towards an origin in the central nervous system (CNS) than the peripheral nervous system, with a crucial role for central sensitization (CS) [4, 7-9]. CS incorporates the process in which the CNS is in a state of hyperexcitability leading to amplified pain sensation (pain hypersensitivity) [9, 10]. Detecting signs of CS can be done with specific psychophysiological tests, also known as Quantitative Sensory Testing (QST), for instance by looking at wind-up, aftersensations or dynamic mechanical allodynia [11]. There is ample evidence that patients with FMS show different signs of CS when assessed with QST [12-20]. Interestingly, one of the hallmark signs of FMS (an increased deep pressure pain sensitivity) has traditionally been considered a sign of peripheral sensitization [21, 22]. More recent studies have, however, shown that deep pressure pain also depicts signs of CS, as it can spread beyond the primary lesion (secondary hyperalgesia) and is correlated to alterations in patients' CNS processing [23-25]. The widespread character of the pressure pain hypersensitivity furthermore indicates an important role for the CNS in the pathophysiology of FMS [21, 26]. An important neurobiological factor for the initiation of CS is the persistent activation of the NMDA receptor throughout different levels of the CNS (dorsal horn, brainstem, basal ganglia, and cerebellum) [4]. The activated NMDA receptors facilitate neuroplastic changes that lead to altered functional connectivity in ascending and descending pain pathways subsequently leading to central pain sensitization symptoms (allodynia and hyperalgesia) [27, 28]. NMDA receptor antagonists (e.g., S(+)-ketamine) have proven to exert an analgesic influence on pain hypersensitivity in FMS [29-34], deeming it a promising pharmaceutical treatment strategy. However, S(+)-ketamine has a strong dose-dependent analgesic effect and higher doses of S(+)-ketamine are associated with an increased severity of side effects [35]. Treatment of FMS with S(+)-ketamine is therefore debated. In the Netherlands, S(+)-ketamine is mainly used in pain clinics to help patients with treatment-resistant chronic refractory pain [36]. In FMS, a lot of patients are also resistant to the current pharmacological treatment strategies [37-40], which warrants alternative pharmaceutical options like S(+)-ketamine [41]. Furthermore, treatment strategies for FMS have shifted towards non-pharmacological approaches in order to increase their efficacy, yet despite these efforts the effect sizes of FMS treatments remain modest [42]. A treatment strategy combining a pharmacological as well as a non-pharmacological approach and thereby maintaining the analgesic effect of S(+)-ketamine, whilst minimizing side-effects, could optimally balance out the benefits and risks of S(+)-ketamine ad-

ministration in FMS. Additionally, such a treatment strategy might strengthen the implementability of S(+)-ketamine administrations in future clinical guidelines since the amount of side-effects currently prevent frequent administration in daily practice [35].

A way of improving the treatment of pain hypersensitivity is by using placebo effects [43, 44]. The placebo effect is described as the possible beneficial effect for a certain outcome in a clinical or laboratory settings due to a persons' neuropsychological mechanisms (e.g., expectancies) when an inert substance is administrated or as part of an active treatment [45, 46]. Placebo effects are influenced through multiple psychological processes, but predominantly by expectations [46, 47]. One way of eliciting these expectations is through pharmacological conditioning, a form of classical conditioning, where the body is conditioned for a response of a medical substance by replacing this substance with a placebo [48]. In practice, pharmacological conditioning forms the basis for dose-extending placebos, which are used to intersperse with active medicine in a dose extending treatment regimen [49]. This incidental substitution of placebos and active medicine reduces the overall dose needed for therapeutic effects and hence may reduce drug-specific side effects [47, 48]. In pharmacological conditioning with S(+)-ketamine, the conditioned analgesic response of the body to a placebo mimicking the effects of ketamine could result in a lower required dose of S(+)-ketamine and resultantly in less dose-dependent side-effects. This new treatment strategy with S(+)-ketamine might therefore be an interesting addition to the current medical treatment of pain sensitization in FMS. However, to our knowledge, a study of pharmacological conditioning with S(+)-ketamine in patients with FMS has never been conducted before.

As mentioned earlier, assessing chronic pain with specific parameters (e.g., psychophysical testing) is a complex process and until now, only subjective markers seem valid enough for use in clinical practice [50]. There is a need for markers that measure pain objectively, especially in chronic pain states, as patients can struggle to communicate their complaints to clinicians [51, 52]. One interesting, yet so far unresearched marker, is respiratory variability. Pain has profound effects on the respiratory system, including an increased respiratory rate, an increased tidal volume, or a combination of both [53]. These respiratory parameters are typically not constant, but show significant fluctuations over time. This is defined as respiratory variability [54]. Variability of respiratory rate and tidal volume has predictive value for a variety of adverse clinical outcomes, such as respiratory failure in critically ill patients [55]. It is also affected by anxiety and complex cognitive tasks [54]. Little is known however about the role of pain in the variability of breathing. Potentially, a physiological parameter such as variability of breathing may prove to be a useful, additional objective marker of chronic pain.

In addition to breathing variability being considered a possible novel marker of pain, assessing the effects of treatments for chronic pain (e.g., S(+)-ketamine) on breathing variability could expand its use from solely a diagnostic tool to a monitoring tool as well. For example, in a recent study investigating the effects of morphine administration in patients with obstructive sleap apnea breathing variability was successfully implemented to monitor treatment effects [56]. The effects of S(+)-ketamine on respiratory variability are poorly understood. In general, S(+)-ketamine induces a sympathico-adrenal activation that may stimulate breathing [57]. In an observation study in patients under procedural sedation with propofol and remifentanil, S(+)-ketamine preserved respiratory variability whereas midazolam decreased variability of respiratory rate and tidal volume [58]. However, little is known about the effects of these medications on respiratory variability in awake patients with chronic pain. We will therefore study the effects of S(+)-ketamine on respiratory variability in patients with FMS.

The following research proposal comprehends a strategy for investigating the central role of pharmacological conditioning with S(+)-ketamine in FMS-related pain hypersensitivity. In this proof-of-principle study, the primary research question is whether pharmacological conditioning with S(+)-ketamine reduces pain hypersensitivity (measured with QST's) in patients with FMS compared to placebo. It is hypothesized that pain hypersensitivity is more effectively reduced by pharmacological conditioning with S(+)-ketamine than placebo conditioning. Additionally, we will study clinical pain intensity, subjective disease impact and side effects of S(+)-ketamine administration. We will also characterize the variability of breathing in patients with FMS continuously undergoing S(+)-ketamine, midazolam and saline administration.

2. OBJECTIVES

<u>Primary Objective:</u>

The primary objective is to investigate whether pharmacological conditioning with S(+)-ketamine compared to pharmacological conditioning with placebo medication reduces pain hypersensitivity in patients with FMS.

<u>Secondary Objective:</u>

The secondary objectives are:

a. To investigate whether pharmacological conditioning with S(+)-ketamine compared to pharmacological conditioning with placebo medication reduces clinical pain intensity in patients with FMS.

b. To investigate whether pharmacological conditioning with S(+)-ketamine reduces subjective disease impact in patients with FMS.
c. To investigate whether pharmacological conditioning with S(+)-ketamine reduces side effects by ketamine in patients with FMS.
d. To investigate the variability of respiratory rate and tidal volume in patients with FMS.
e. To investigate whether S(+)-ketamine increases the variability of respiratory rate and tidal volume in patients with FMS.
f. To investigate whether midazolam decreases the variability of respiratory rate and tidal volume in patients with FMS.
g. To investigate whether induced pain during Quantitative Sensory Testing increases the variability of respiratory rate in patients with FMS.

3. STUDY DESIGN

The following proof-of-principle trial is an intervention study with a double-blinded, randomized placebo controlled design. Participants are initially allocated to one of three groups: 1) S(+)-ketamine conditioning , 2) active placebo (midazolam) conditioning and 3) passive placebo (saline) conditioning. Similar to previous pharmacological conditioning studies, the conditioning paradigm will consist of two phases: the acquisition phase and the evocation phase. In the acquisition phase, which will last 3 weeks in total, participants will be asked to visit the hospital once a week to receive either an active therapy, in the form of IV S(+)-ketamine, or a placebo therapy, which is either active (IV midazolam) or passive (IV saline). Along with the IV administration, participants in all groups will receive a verbal suggestion that they are receiving either a strong painkiller or a placebo. Neither the participant nor the experimenter knows which intervention the participants receive. In the evocation phase, which starts after the third week of acquisition (on day 22), and lasts for another week, participants will visit the hospital once and receive IV saline. The participants are also verbally instructed with the same verbal suggestions as before (either receiving a strong painkiller or placebo). Informing participants about the study medication in the exact same manner as done in the acquisition phase is essential for pharmacological conditioning. The information given during the procedure acts as a neutral stimulus that becomes a conditioned stimulus in a conditioning paradigm [49]. Together with other contextual factors, the verbal instructions form the conditioned stimuli that elicit the conditioned response in pharmacological conditioning [59]. Changing verbal instructions might impact the conditioned response significantly. In addition, openly sharing with participants that they receive a sham infusion might induce negative expectations or nocebo effects. As shown by a previous study [59], negative expectations can attenuate or even completely reverse the effect of pharmacological conditioning in pain. The application of a covertly administrated placebo is

thus favored in the protocol. At the end of the study, participants will be extensively debriefed about pharmacological conditioning and the necessity for deception. For an overview of the design, see Figure 1.

The study outcomes will be assessed in the acquisition and evocation phase to optimize blinding for participants and prevent response bias. Outcomes are obtained at baseline, during, and after receiving the intravenous administrations of either S(+)-ketamine, midazolam or saline. The conditioned effects on pain sensitivity are assessed by analyzing three different QST modalities: 1) pressure pain thresholds (PPT's), 2) temporal summation or wind-up pain, and 3) aftersensations. The QST modalities are studied with pressure stimuli applied onto two different body locations: hand and lower leg. The primary endpoint chosen for this study is the conditioned effects of S(+)-ketamine on pressure pain thresholds. As shown in previous studies, the pressure pain threshold is a reliable and valid static QST modality to study amplified pain sensitivity in patients with FMS [23, 24, 60]. The conditioned effects of the S(+)-ketamine group are investigated by subtracting the PPT's collected at baseline from the scores collected directly post-intervention in the evocation phase and comparing these with the placebo groups. The secondary endpoints wind-up pain and aftersensations are simultaneously obtained with PPT's to study pain sensitivity more specific to central processing. A possible influence of body location on conditioned QST effects is also secondarily examined. Clinical pain intensity scores are obtained with the collection of QSTs to assess the conditioned effects of the interventions on chronic widespread pain [61]. Another secondary endpoint is extinction of pharmacological conditioning, which is studied by looking at the size of the difference scores over time [62]. Evaluation of subjective disease impact of FMS will be studied with the Fibromyalgia Impact Questionnaire – Revised (FIQR), which is collected at baseline and after one week follow-up in the acquisition and evocation phase [63]. Side effects of the study medications will be investigated by looking at vital signs (heart rate, blood pressure, breathing rate, oxygen saturation, and temperature), assessment of sedation levels, presence of nausea or vomiting, and assessment of the Bowdle Questionnaire [64]. Vital signs are obtained to study sympathomimetic side effects (e.g., hypertension and tachycardia). Sedation level is assessed with a Ramsay score and used for monitoring central nervous system depression or hyper excitation [65]. Assessing nausea and vomiting is necessary to monitor the vestibular perturbations that can be caused by S(+)-ketamine [66]. The Bowdle Questionnaire is used to measure psychosomatic side effects and outcomes will be collected simultaneously with pain assessments (clinical pain intensity and QSTs) [67].

Respiratory rate, changes in tidal volume and changes in minute ventilation are measured continuously at baseline, during, and after receiving the intravenous administrations of either S(+)-ketamine, midazolam or saline. These measurements are performed using an

impedance-based superficial respiratory volume monitor (ExSpiron, Respiratory Motion, Walthan, MA, US) with a thoracic electrode [68].

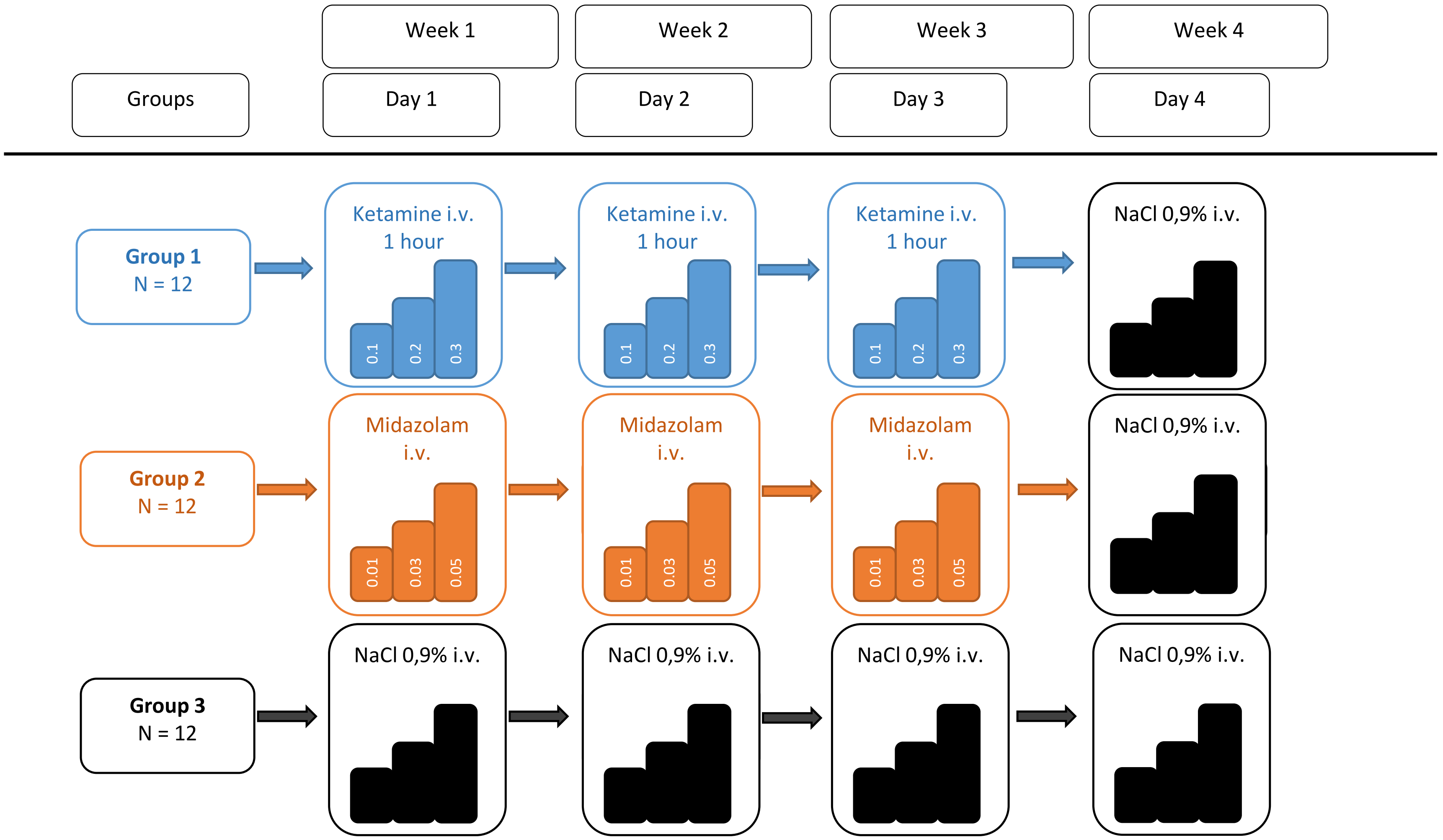

Fiqure 1 Diagram of study design. Acquisition phase = week 1,2, and 3. Testing phase = week 4. The administration of medication is increased over time in accordance with the step-up protocol for the study.

4. STUDY POPULATION

4.1 Population (base)

Participants will be 36 female fibromyalgia patients, contacted via patient organizations and treatment centers in the Netherlands, as well as via (social) media, such as Facebook or local newspapers.

4.2 Inclusion criteria

In order to be eligible to participate in this study, a participant must meet all of the following criteria:

- Age between 18 and 75 years old
- Female gender
- Diagnosed with FMS by a rheumatologist
- Able to understand and speak Dutch

4.3 Exclusion criteria

A potential subject who meets any of the following criteria will be excluded from participation in this study:

- A medical diagnosis other than fibromyalgia explaining the chronic pain symptoms.
- Presence of hypertension or any other severe cardiovascular co-morbidity (e.g., heart failure (NYHA III or IV), neuromuscular diseases, pulmonary obstructive or restrictive diseases, kidney failure (eGFR ≤ 60), liver diseases or epilepsy)
- Presence of any severe psychiatric disease not related to symptoms of FMS (e.g., schizophrenia, schizoaffective disorders, severe anxiety or depression (HADS > 15), bipolar disorder, dissociative personality disorder, or (previous) addiction to strong analgesics.
- Presence of any allergy for S(+)-ketamine, midazolam, ondansetron or flumazenil
- Long term use of medicine that is contra-indicated when administering S(+)-ketamine or midazolam: NMDA-receptor antagonists, parasympathomimetics (e.g., bronchodilators), thyroid hormones, vasopressin, CYP3A4 liver enzyme inhibitors (e.g., verapamil, diltiazem, or certain antibiotics), CYP3A4 liver enzyme inductors (e.g., rifampicin, carbamazepine, fenytoin), and **strong opioids** (e.g., morphine, fentanyl, heroin)
- Previous experience(s) with S(+)-ketamine in a medical setting or previous experience with recreational racemic Ketamine use.
- Use of painkillers different than usual dose of treatment on **the day of experimentation.**

- Use of alcohol or drugs 24 hours prior to the hospital visits
- Use of caffeine 12 hours prior to the hospital visits
- Body weight > 100kg or BMI >35
- Presence of pregnancy or lactation
- Presence of an ICD, pacemaker or implanted medication pump
- Presence of chronic pain at the local site of experimental pain stimuli or monitoring devices.
- Implanted materials in either arm (e.g., non-removable piercings)

4.4 Sample size calculation

Sample size calculation of the totally needed amount of participants was based upon a previous pharmacological conditioning study [69]. Although not fully comparable, the study addresses non-opioid pharmacological conditioning with Ketorolac, which might induce similar effects to conditioning with S(+)-ketamine. A calculated Cohen's d for the effect between the natural history group and ketorolac conditioning group was 1.75, which equals a partial eta squared of 0.4336. Due to planned contrasts an $\alpha = 0.025$ was chosen along with a power of 80% ($\beta = 0.8$). G*Power version 3.1.9.4. was utilized to calculate the required sample size and a between-within repeated measures ANOVA model was chosen as the desired analysis method. Although statistical testing in this study will be done with linear mixed models, G*Power is not able to conduct sample size calculations for multilevel modelling and the between-within repeated measures ANOVA is mathematically identical to the required linear model of the main analysis. The main analysis composes three groups (S(+)-ketamine conditioning, midazolam conditioning and saline conditioning), and two measurements over time (baseline and post-intervention). The influence of body location is not incorporated in the primary model since a previous study with ketamine depicted minimal differences [33]. In G*Power, the effect size specification 'as in Cohen (1988)' was chosen to account for correlation among repeated measurements [70]. Running the software yielded a total required sample of 21 participants [71]. As the effect size obtained from the study by Benedetti et al. 2011 was high, we conducted a a more conservative sample size calculation based on a moderate effect size (partial eta squared = 0.06), according to Cohen's 'rule of thumb' and this yielded a total required sample of 51 participants. In this sample size calculation the effect size specification 'as in G*Power 3.0' was chosen as this calculation is not based on previous results [70]. For the current proof-of-principle study, a sample size in between these estimations was chosen, including 10 participants per study group. The drop-out margin is anticipated to be substantial (16.7%) as a result of the study medication and thus a total of 2 participants **per study group** will be included for drop-outs. The total number of required participants is 36.

5. TREATMENT OF SUBJECTS

This study investigates pharmacological conditioning with S(+)-ketamine in patients with FMS. The intervention group will be compared to a placebo group of patients that will receive either midazolam conditioning (active placebo) or saline conditioning (passive placebo). The analgesic effects due to pharmacological conditioning are investigated with the infusion of IV saline. The study takes place at the outpatient clinics of the department of anaesthesiology at the Amsterdam University Medical Center (AUMC), location VUmc. Due to the fact that participants in this study are subjected to sedative medication, the protocol has been reviewed and approved by the sedation committee of the AUMC. As such, the experiment will adhere to the safety regulations and specific sedation protocols of the AUMC.

5.1 Investigational product/treatment

S(+)-Ketamine

Ketamine is a pharmaceutical agent, which belongs to the NMDA receptor antagonists. It is commonly used for either the induction and maintenance of anesthesia, or the treatment of pain [72]. Ketamine's anesthetic and analgesic effects are mainly attributable to the blockade of NMDA receptors, which prevents the excitation of the central nervous system from glutamate [66]. Ketamine has a chemical structure which contains two optical isomers: R(-)-ketamine and S(+)-ketamine. The S(+)-enantiomer is approximately three to four times more potent analgesic and anesthetic than the racemic mixture or R(-)-enantiomer [35]. Therefore, the same beneficial effects are achieved with a smaller dosage, while less side effects are experienced by participants. Anesthesia with S(+)-ketamine is commonly induced with a bolus administration of approximately 0.5 – 1mg/kg and maintained with a continuous infusion rate of 0.5 – 3mg/kg/hour. Analgesia is established with a bolus of 0.1 – 0.25mg/kg and maintained with a continuous infusion rate of 0.2 – 1mg/kg/h [73]. In the present study, pharmacological conditioning of NMDA receptor antagonists will be investigated with the S(+) enantiomer of ketamine due to its beneficial profile. The drug will be infused intravenously for one hour with a step-up dose regimen of 0.1mg/kg/h – 0.2mg/kg/h – 0.3mg/kg/h. The step-up dose regimen is used to assess the appropriate dose for a participant, which is based upon the toleration of side effects and the benefit of analgesic effects [74, 75]. The infusions of IV S(+)-ketamine are administered once a week during every administration day in the acquisition phase (see Fig. 1).

Active Placebo

In the current study, midazolam will serve as an active placebo since successful blinding for the psychotomimetic effects of S(+)-ketamine is less reliable when only using a passive placebo (IV saline) [74, 76]. Midazolam is an anesthetic that causes sedation and confusion without antinociception, which deems it an appropriate placebo in pain studies

[77]. In a similar study investigating the effects of S(+)-ketamine in patients with FMS, midazolam was used as an active placebo and indeed led to an adequate blinding of the participants [64]. The effects of midazolam on chronic pain have to be considered carefully though as there is conflicting evidence regarding the influence of benzodiazepines on central sensitization [78-82]. Therefore, to prevent any unwanted analgesic effects in the control group, a second (passive) placebo group is added to this study. The interventions in this passive placebo group are described below. In the active placebo group, IV midazolam will be infused for one hour in a step-up dose regimen of 0.017 – 0.033 – 0.050mg/kg/h, similar to the S(+)-ketamine group, during every administration day in the acquisition phase (see Fig. 1).

<u>Passive placebo</u>

In this study, IV saline will serve as a passive placebo. IV saline will be administered over the course of one hour during every administration day in the acquisition and evocation phase. Due to the isotonic characteristics, few side effects in low doses, easy administration, and low costs, saline is regularly used for sham infusions in research [59, 76, 83-85].

5.1 Use of co-intervention (if applicable)

Patients with FMS are allowed to continue their usual medication, but changes in medication will not be allowed as of a month prior to testing to prevent influence of usual care during testing. The following pain medication will be allowed throughout the study: acetaminophen, non-steroidal anti-inflammatory drugs (NSAID’s), selective serotonin inhibitors (SSRI’s), tramadol, tricyclic antidepressants (TCA’s), and pregabalin or gabapentin. This pain medication has been used safely in a previous study that assessed the influence of S(+)-ketamine [75].

5.2 Escape medication (if applicable)

In case of unbearable pain, subjects are allowed to use escape medication during the acquisition and evocation phase, except for the day of experimentation. The escape medication consists of acetaminophen and NSAIDs (for example ibuprofen, diclofenac). In the case of acetaminophen, participants are allowed to use a maximum of 1000 milligram (two tablets of 500 milligram) for four times a day, which makes a total maximum dose of 4000 milligrams per day. In the case of ibuprofen, participants are allowed to use a maximum of 400mg for three times a day, which makes a total maximum dose of 1200 milligrams per day.

In case of severe anxiety after S(+)-ketamine administration in the hospital, the infusion of S(+)-ketamine is halted and if necessary, an additional bolus of IV midazolam 2mg will be

used to treat the symptoms. Due to the rapid metabolization of S(+)-ketamine, it is expected that any side effects will however resolve shortly after its cessation [74, 86]. If a bolus of midazolam is necessary, this will be authorized by an on-call anesthesiologist at the Amsterdam UMC and the injection will be administered into the IV line by medical staff involved in the research.

In case of persistent nausea or vomiting after ketamine administration in the hospital, a bolus of IV ondansetron 4mg (an antiemetic) will be used to treat nausea and prevent vomiting. This will be authorized by an on-call anesthesiologist at the Amsterdam UMC and the injection will be administered into the IV line by medical staff involved in the research.

In case of prolonged apneas during midazolam infusion, the infusion of midazolam will be halted and an injection of IV flumazenil 0.2mg (a benzodiazepine antagonist) will be administered. This will be authorized by an on-call anesthesiologist at the Amsterdam UMC and the injection will be administered into the IV line by medical staff involved in the research.

6. INVESTIGATIONAL PRODUCT

6.1 Name and description of investigational product(s)

S(+)-Ketamine:

S(+)-ketamine hydrochloride is the name of the 5 mg/ml solution contained in ampoules, corresponding to 5 mg of free S(+)-ketamine base per ml. S(+)-ketamine, the (+) enantiomer of ketamine racemate, belongs to the pharmacotherapeutic group of anesthetics, with ATC-code N01AX03. Ketanest-S solution for injection is registered under RVG 22550 and the product information is registered on the CBG website. S(+)-ketamine belongs to the class of organic compounds known as chlorobenzenes (compounds containing one or more chlorine atoms attached to a benzene moiety).

Midazolam:

Midazolam is a pharmaceutical agent that belongs to the class of imidazobenzodiazepines and also a member of monofluorobenzenes. It is commonly used for sedation, hypnosis, decreased anxiety, anterograde amnesia, centrally mediated muscle relaxation and anti-convulsant activity [87]. Benzodiazepines exert their inhibitory effects by potentiating the

effect of GABA on the GABA-A receptor. The effect of benzodiazepines is dose-related: at low doses it protects against anxiety, while at higher doses the effect can induce sedation or hypnosis [87]. Midazolam hydrochloride is the name of the 1mg/ml solution contained in ampoules, corresponding to 1mg of free midazolam base per ml. Midazolam belongs to the pharmacotherapeutic group of anesthetics, with ATC-code N05CD08. Midazolam Aurobindo solution for injection is registered under RVG 22594 and the product information is registerd on the CBG website.

Sodium chloride 0.9% (saline):

Sodium chloride 0.9% or saline is the name of the sterile infusion fluid packaged in 500ml bags, which contains 9 grams of sodium chloride salt per liter or 9mg/ml. Sodium chloride 0.9% belongs to the crystalloid fluids that consist of water solutions with small inorganic ions and small organic molecules [88]. It is commonly used for fluid-depleted patients, dis-solving of medication for intravenous application, or cleansing of medical materials [88]. Sodium chloride has the ATC code B05XA03. Sodium chloride 0.9% is registered under RVG 55227 and the product information is registered on the CBG website.

6.2 Summary of findings from non-clinical studies

S(+)-ketamine:

See SPC (‘D2. SPC Ketanest’) and product information (‘D2.2 Ketanest Bijsluiter’).

Midazolam:

See SPC (‘D2. SPC Midazolam Aurobindo’) and product information (‘D2.2 Midazolam Aurobindo Bijsluiter’).

Sodium chloride 0.9% (saline)

See SPC (‘D2. SPC Natriumchloride’) and product information (‘D2.2 Natriumchloride Bijsluiter’).

6.3 Summary of findings from clinical studies

S(+)-ketamine:

See SPC (‘D2. SPC Ketanest’) and product information (‘D2.2 Ketanest Bijsluiter’).

Midazolam:

See SPC (‘D2. SPC Midazolam Aurobindo’) and product information (‘D2.2 Midazolam Aurobindo Bijsluiter’).

Sodium chloride 0.9% (saline)

See SPC (‘D2. SPC Natriumchloride’) and product information (‘D2.2 Natriumchloride Bijsluiter’).

6.4 Summary of known and potential risks and benefits

S(+)-ketamine:

Ketamine hydrochloride is a cyclohexanone derivative and a selective NMDA receptor antagonist that is used for induction of anesthesia. Common side effects of ketamine are: dissociation, psychotomimetic effects, dysphoria, dizziness, headache, nausea and vomiting, and sympathomimetic effects (tachycardia and hypertension) [66, 89]. Physiological side effects of ketamine (nausea, vomiting, headache, dizziness, tachycardia, hypertension, and increased oxygen saturation) are dose-dependent and most predominant at the dosages used for the induction and maintenance of anesthesia. Psychological side effects (e.g., dissociation, hallucinations, and dysphoria) seem to be less dose-dependent [35, 90], although a clear relationship between dose and ‘drug high’ has been observed in previously executed pharmacological trials [64, 74, 86, 91]. Allergy to ketamine has been reported, but is extremely rare, and will be included as an exclusion criterion in the screening of participants. Ketamine is a racemic mixture consisting of two enantiomers, R(-) and S(+)-ketamine. In contrast to racemic ketamine, S(+)-ketamine is reported to be less likely to evoke psychological side effects, although evidence for this is conflicting [91, 92]. S(+)-ketamine is also used as a general anesthetic (bolus administration of approximately 1mg/kg and infusion of approximately 0.5mg/kg/hour + midazolam infusion) and has been used at subanesthetic doses in trials studying the treatment and basic science of pain and depression [35, 73, 93, 94]. Studies utilizing subanesthetic S(+)-ketamine in continuous intravenous infusions have used dosages ranging from 0.1 to 0.57mg/kg [64, 75, 76, 86, 95-98]. The main side effects reported were nausea and vomiting, dizziness, sedation, or hallucinations. They were often well tolerated and cessation of participation due to their severity was very rarely observed (only in one study [76]). Studies investigating the effects of ketamine or S(+)-ketamine in patients with FMS have shown that significant analgesia can be achieved without the occurrence of severe side effects [31, 33, 64]. The predominant side effects reported were: dizziness, impairment of position, hearing impairment, and ‘drug high’. Although severe anxiety/dysphoria has not been reported in any study investigating patients with FMS receiving ketamine, a careful dosing approach will be implemented in the current study due to the high incidence of anxiety symptoms in this patient category [3]. Given the results of the above mentioned studies, a dose between 0.1 – 0.3mg/kg/h will pose a minimal risk for the participants in this study. In order

to individually assess how well side effects are tolerated, a step-up dose regimen will be implemented in this study, as well as a health and psychiatric screening of participants (see also section 6.5).

<u>Midazolam:</u>
Midazolam hydrochloride is a short-acting benzodiazepine derivative with an imidazole structure. It has been shown to act as a GABA-A agonist that causes inhibition in the central nervous system. The most common psychological side effects for benzodiazepines are: drowsiness, apathy, disorientation, confusion, headache, depression, visual disturbances. These side-effects are mostly reported when anesthetic doses (0.1 – 0.3 mg/kg) of midazolam are given and are clearly dose-related [87, 99]. Another important psychological effect from midazolam is the paradoxical phenomenon of agitation and hallucinations, which can occur in 16,7% of patients when given a sedative dose (0.1mg/kg) [100]. Fortunately, this phenomenon can easily be reversed with a single dose (0.2mg/kg) of a GABA-A antagonist (i.e. Flumazenil). Physiological side effects of midazolam are mainly cardiovascular, respiratory, and gastro-intestinal. Cardiovascular effects are limited to hypotension and reflex tachycardia due to a loss in systemic vascular resistence, occurring mainly during a loading dose of midazolam (0.15mg/kg) [101]. Respiratory effects of midazolam are threefold: it reduces the respiratory response to CO^2, it causes weakening of muscular tone leading to upper airway obstruction and it induces respiratory depression [87]. Respiratory effects are likely to occur at a loading dose of ≥ 0.07mg/kg, although this is conflicted by some studies [102-105]. In studies using continuous infusions of midazolam, apnea or hypoxemia was not seen at doses between 1-5mg/h (~ 0.01 – 0.07mg/kg/h) [64, 106-108], even though the study with low-dose (0.01mg/kg/h) midazolam also administered a loading dose of 0.5mg [106]. Common gastro-intestinal side effects for midazolam are: nausea and hiccups [109]. Allergic reactions to midazolam have been reported in various frequencies, but the overall incidence is low and mostly limited to peri-procedural reactions [110]. Studies investigating benzodiazepines in patients with FMS are limited and have either looked at their effectiveness on pain or have used them as active placebo's [64, 80, 81]. In the study by Noppers et al., an 0.5h continuous infusion of 5mg (~ 0.13mg/kg/h) of IV midazolam was used as an active placebo and was well tolerated as none of the patients dropped out due to side effects [64]. In two earlier studies, which compared the effects of oral alprazolam, and ibuprofen to placebo, 3 of the 65 patients did not complete the study protocol, mainly because of drowsiness complaints. However, it has to be noted that at least 4 patients of the placebo groups also dropped out due to side effects, which questions what the true influence of benzodiazepines on the likelihood of a drop-out was

[80, 81]. Given the results of above mentioned studies, a dose between 0.01 – 0.05mg/kg/h will pose a minimal risk for the participants in this study. Similar to the ketamine administration, participants receiving midazolam will go through a step-up dose regimen to observe their toleration of side effects, and will receive a health and psychiatric screening.

<u>Sodium chloride 0.9% (saline):</u>

Sodium chloride belongs to the crystalloid fluids and contains 154mmol/L of sodium and equal amounts of chloride. It is predominantly used as a resuscitation fluid, solution for intravenous medication, or cleansing of medical material. As a consequence of its composition, the most frequent reported side-effects are hyperchloremia, metabolic acidosis, and renal failure [88]. However, these side-effects are observed when participants receive large amounts of sodiumchloride (2 liter in 1 hour, ~ 30ml/kg/h) [111]. In an Italian cross-sectional study, no serious adverse events were observed in patients receiving an average of 1000ml – 1300ml of intravenous fluids per day [112]. Therefore, the risks of infusion of 50ml of saline in 1 hour are considered nihil for this study.

6.5 Description and justification of route of administration and dosage

<u>S(+)-Ketamine:</u>

S(+)-ketamine is commonly administered via a continuous IV pump-infusion. When ketamine, or one of its' enantiomers, is taken orally it is not broken down by the stomach acids, but rather it is absorbed in the intestine unchanged. From there, it is carried to the liver where it is immediately metabolized into metabolites that can increase mental and cardiac effects. These metabolites (e.g., norketamine) then enter the bloodstream from where it reaches the rest of the body. Thus, oral ketamine is less effective than IV administration and increases undesirable side effects [113]. When giving ketamine intravenously, the amount and timing of the medicine is more quickly and precisely controlled and the appropriate effects can be achieved more accurately [114]. Peak plasma concentrations after IV administration are reached within 2 to 10 minutes [115]. Effective analgesic concentrations of S(+)-ketamine seem to be reached at 70-100ng/ml, corresponding to a dose of 0.3mg/kg/h [75, 86], although clinical effects differ between studies, with some of them already reporting significant analgesia after 0.15mg/kg/h of S(+)-ketamine [96]. Infusion of IV S(+)-ketamine is commonly and safely used in clinical practice for the induction of anesthesia and analgesia, including in vulnerable populations such as elderly postoperative patients [116]. Also, infusion of IV S(+)-ketamine has been administered in patients with FMS without the occurrence of major side effects [64]. Studies have however shown that side effects due to S(+)-ketamine administration can start at a low

plasma concentration (<100ng/ml), and safe administration is thus warranted [86, 117]. In order to safely administer S(+)-ketamine, a step-up dose regimen of 0.1 – 0.2 – 0.3mg/kg/h during one hour is used in this study. More specifically, participants will be individually assessed for analgesic effects and side effects after every twenty minutes and only if side effects are well tolerated, the dose of S(+)-ketamine will be increased with Δ0.1mg/kg/h. If side effects are not well tolerated at 0.1mg/kg/h, the infusion is stopped and the pt. will be excluded from the analysis. If side effects are not well tolerated at 0.2mg/kg/h, but are tolerated at 0.1mg/kg/h and the clinical and experimental pain is relieved, than the infusion is continued for the remaining 40 minutes with a dose of 0.1mg/kg/h. The same situation holds when side effects are not well tolerated at 0.3mg/kg/h (see fig. 2). A comparable regimen of 0.05 – 0.1 – 0.15 mg/kg/h in 1.5 hours has been used in a previous study and resulted in an effective and safe administration of S(+)-ketamine [96]. The step-up dose regimen of 0.1 – 0.2 – 0.3 mg/kg/h in the current study will result in a total of 14mg/70kg/1h of IV S(+)-ketamine, which has also proved to be an effective and safe dosage in previous studies [35, 98]. It is therefore anticipated that the current step-up dose regimen is the preferred dose scheme in the studied patient population. Reaching the required dose at the predefined time intervals is done by increasing the infusion flow (ml/h) of the study medication pumps by the primary researcher (see D6.2 KetCOP studie infusieschema).

<u>Midazolam:</u>

Administration of midazolam occurs very often via a continuous IV pump-infusion and is in clinical practice most often used for the induction of anesthesia or maintenance of sedation. Midazolam has few side effects compared to other sedatives [118]. Anesthesia is commonly induced with a bolus administration of approximately 0.3mg/kg and maintained with a dose of approximately 0.03 – 0.1 mg/kg [87, 119, 120]. Conscious sedation can be achieved with a dose varying between 0.025 – 0.07mg/kg/h [64, 121]. In contrast to S(+)-ketamine, midazolam is absorbed rapidly by the gastro-intestinal tract and transported to the liver, where it is rapidly metabolized to its inactive forms. Due to this rapid hepatic clearance, the bio-availability of oral Midazolam is 40-50%, which means that an oral dose has to be approximately twice as high as an intravenous dose to achieve similar effects [101]. Additionally, the onset of clinical symptoms after oral administration is approximately 1 hour, which is comparable to its oral peak plasma time and significantly longer than onset of symptoms when given intravenously, which is 30-60 seconds [87]. This delay in onset expands the time of the current experiment, which is undesirable for study participants and comparability between groups. Also, infusion of IV midazolam (or other

benzodiazepines) has been safely conducted in patients with FMS [64, 80-82]. The infusion of IV midazolam is executed similarly to the infusion of S(+)-ketamine to optimally blind the experiment. This translates to a step-up dose regimen of 0.017 – 0.033 – 0.05mg/kg/h over the course of one hour. Similar to the S(+)-ketamine administration, toleration of side effects will be assessed during and after every step-up (20 minutes) in the dosing scheme and the midazolam dose is only increased if side effects are well tolerated (see Fig. 2). The total amount of midazolam for a one hour administration equals to 2.33mg/70kg. Based on the previous literature, this is an effective and safe dosage for purposes of this 1 hour active placebo administration.

<u>Sodium chloride 0.9% (saline):</u>
Administration of sodium chloride 0.9% can occur in various ways; continuous infusion, bolus infusion, or intranasal administration [88, 122]. The amount of sodium chloride dispersed in this study will be 9mg * 50 ml = 450mg or 154 mmol * 0.05L = 7.7mmol/L for the purpose of it being comparable to the study medication. The infusion flow (ml/h) of the saline pumps will be increased at similar rates as the infusion flow of the S(+)-ketamine and midazolam pumps to ensure optimal blinding of in this group (see D6.2 KetCOP infusieschema).

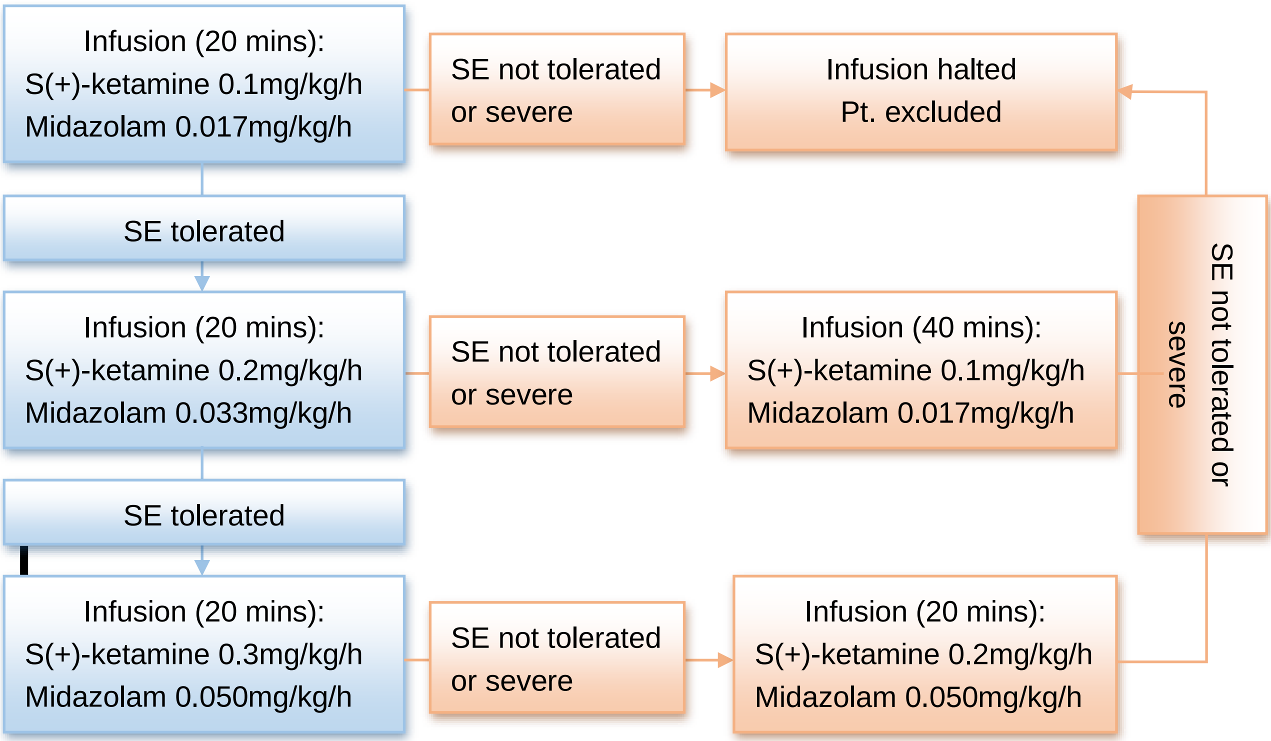

Fig. 2. Dosing diagram of study: during every increment, side effects (SE) are assessed and if participants do not tolerate them or they are too severe for continuation of the infusion, the infusion is either halted or the administered dose is lowered.

6.6 Dosages, dosage modifications and method of administration

In this study, the pharmacy of the LUMC and Amsterdam UMC will co-operate to facilitate study medication. In the LUMC, study medication is labelled according to GMP guidelines (annex 13) after which it is transported to the Amsterdam UMC pharmacy. For blinding reasons, the Amsterdam UMC pharmacy will dispend re-packaged and re-labelled IV-solution syringes containing ketamine, midazolam, or saline. Personnel from the pharmacy will also randomize participants across groups using CastorEDC (see also section 8.2). Connection of IV-pumps to lines and placement of vascular catheters and access ports will be performed by trained medical staff involved in this research.

<u>S(+)-ketamine:</u>

A continuous step-up dose IV pump-infusion of S(+)-ketamine will be administered for 1 hour with the following incremental steps; 0.1mg/kg/h for 20 minutes, followed by 0.2mg/kg/h for 20 minutes followed by 0.3mg/kg/h for 20 minutes. The required dose for every 20 minutes during the hour of administration is reached by adjusting the pump flow (ml/h). The pump flow increments used in this study are: 25ml/h – 50ml/h – 75ml/h, adjusted every 20 minutes. See also D6.2 KetCOP studie infusieschema. The administration will start after baseline measurements.

<u>Midazolam:</u>

A continuous step-up dose IV pump-infusion of midazolam will be administered for 1 hour with the following increments; 0.017mg/kg/h for 20 minutes, followed by 0.033mg/kg/h for 20 minutes, followed by 0.05mg/kg/h for 20 minutes. The required dose for every 20 minutes during the hour of administration is reached by adjusting the pump flow (ml/h). See also D6.2. KetCOP studie infusieschema. The administration will start after baseline measurements, see also section 8.3.

<u>Sodium chloride 0.9% (saline):</u>

A continuous IV pump-infusion of saline will be administered for 1 hour. The flow of the saline pump is increased every 20 minutes and follows the infusion speeds of the S(+)-ketamine, and midazolam groups. See also D6.2 KetCOP studie infusieschema. The administration will start after baseline measurements.

6.7 Preparation and labelling of Investigational Medicinal Product

<u>S(+)-ketamine:</u>

S(+)-ketamine is prepared and GMP labelled by the LUMC pharmacy. The LUMC pharmacy has previously prepared S(+)-ketamine IV bags for comparable studies [64]. See in appendix SPCs (‘D2. SPC Ketanest’ and ‘D2. SPC Midazolam’) and D3.Voorbeeldetiketten KetCOP. The medication will then be transported to the pharmacy of the Amsterdam UMC, location VUmc via WorldCourier. This pharmacy will re-package, re-label, and dispend the medication in 50ml syringes (see also section 6.6).

<u>Midazolam:</u>
Midazolam is prepared and labelled by the LUMC pharmacy. The LUMC pharmacy has previously prepared midazolam IV bags for comparable studies [64]. See in appendix SPCs (‘D2. SPC Midazolam’) and D3.Voorbeeldetiketten KetCOP. Similar to the S(+)-ketamine, the midazolam will be transported to the Amsterdam UMC where it will be re-packaged, re-labelled and dispended for the experiment.

<u>Sodium chloride 0.9% (saline):</u>
Sodium chloride infusions are prepared at the Amsterdam UMC, location VUmc pharmacy. See in appendix SPCs (‘D2. SPC Natriumchloride’).

6.8 Drug accountability

Not applicable

7. NON-INVESTIGATIONAL PRODUCT

7.1 Name and description of non-investigational product(s)

Not applicable

7.2 Summary of findings from non-clinical studies

Not applicable

7.3 Summary of findings from clinical studies

Not applicable

7.4 Summary of known and potential risks and benefits

<*See explanatory text of chapter 7.2, including remark*>

Not applicable

7.5 Description and justification of route of administration and dosage

Not applicable

7.6 Dosages, dosage modifications and method of administration

Not applicable

7.7 Preparation and labelling of Non Investigational Medicinal Product

Not applicable

7.8 Drug accountability

Not applicable

8. METHODS

8.1 Study parameters/endpoints

8.1.1 Main study parameter/endpoint

The main goal for this study is to assess changes in pain sensitivity due to pharmacological conditioning with S(+)-ketamine compared to pharmacological conditioning with (combined) placebo medication. Pain sensitivity is quantified in this study by investigating three different Quantitative Sensory Testing (QST) modalities; pain pressure thresholds (PPT's), pressure wind-up pain and aftersensations. The QSTs in this study are executed with pressure stimuli (in kg) applied with a handheld analogue algometer capable of delivering 10kg of force (Force Dial; Wagner Instruments, Greenwhich CT, USA, see also D6.3 Manual Algometer). Pressure pain stimuli seem to be the most adequate method of QST application in patients with FMS as they correlate better to their clinical pain then for example heat pain stimuli, correlate to their CNS hyperexcitability (by means of increased glutamate levels) and respond well to ketamine treatment [23, 24, 26, 31, 33, 123, 124]. The pressure stimuli are applied to the thenar muscles of the dominant hand and ipsilateral tibialis anterior muscle. These muscle areas are preferred because they can accurately display any signs of pain sensitivity, have shown acceptable reliability, are known to be sensitive to ketamine treatment, and are also accessible during testing [31, 33, 60, 125, 126]. PPT's are the main parameter for this study. The main endpoint is a difference between PPT levels at baseline and directly post-intervention (at T = 60mins) between the study groups. The PPT's are examined by applying three

different pressure stimuli (kg force) at three adjacent areas on the hand and lower leg. The three pressure stimuli necessary to evoke pain are then averaged out to calculate a mean pressure threshold in Kg force [127]. The mean pressure thresholds are subsequently averaged out for body location, as a minimum role for body location is expected. PPT's are preferred as the main parameter in this study as they:

1) Are able to reliably demonstrate pain hypersensitivity (due to central or peripheral causes) in patients with FMS [1, 2, 20, 26, 30, 33].

2) Can always be defined.

3) Have shown good to excellent reliability in healthy controls (ICC > 0.7) as well as in patients with FMS (ICC = 0.85) [128, 129].

4) Are increased by S(+)-ketamine administration [30, 32, 33]. PPT's therefore seem to be a valid way of testing the analgesic effect of S(+)-ketamine on pain sensitivity in patients with FMS.

Hence, PPT's are chosen as the primary measurement method for studying the effects of conditioning S(+)-ketamine on pain sensitivity in this study.

8.1.2 Secondary study parameters/endpoints (if applicable)

The secondary endpoints are:

1. The conditioned effects of S(+)-ketamine on wind-up pain measured as the change from baseline and compared to the conditioned effects in the placebo groups. Wind-up pain is a physiological increase in pain in response to a sequential train of equal pain stimuli [9]. The pain hypersensitivity is caused by repetitive C-fiber activation, which leads to an increased responsiveness of dorsal horn neurons (Wide Dynamic Range) to every successive stimulus [130]. Wind-up pain is an important process in central sensitization [9, 131] and studies investigating patients with FMS have shown that wind-up is often enhanced [1, 2, 17, 19]. The phenomenon of wind-up pain can be measured with temporal summation, a dynamic QST modality. Temporal summation is assessed by applying pressure pain at pain threshold level while asking participants to rate the 1st, 5th, and 10th stimulus on an NRS scale [127]. A single temporal summation or wind-up pain score is then obtained by subtracting the 1st stimulus from the 10th (final) stimulus [1, 2]. The wind-up pain is assessed at two body locations: the hand and lower leg and the wind-up pain score will be averaged out for both locations as a minimal influence is expected. Importantly, S(+)-ketamine is able to alleviate wind-up pain in patients with FMS [31]. Reliability of pressure temporal summation was investigated in musculoskeletal

trauma patients and was moderate to excellent (ICC range 0.69–0.91) [60]. Temporal summation is a valid measure for detecting signs of central pain sensitivity and is therefore used in this study.

2. The conditioned effects of S(+)-ketamine on aftersensations measured as the change from baseline and compared to the conditioned effects in the placebo groups. The aftersensations are obtained by asking a participant to rate late pain (in NRS) 15 seconds after the 10th summation stimulus on the hand and lower leg and subtracting this pain score from the 10th score of the summation sequence. Similar to the pressure pain thresholds and wind-up pain, the scores for aftersensation of the hand and lower leg will be averaged out. Although the relation between painful aftersensations and CS has been studied to a lesser extent then temporal summation, evidence from previous studies has indicated that CS enhances aftersensation levels [15, 17].

3. The influence of body location on the QST's (PPT, temporal summation, and aftersensations). Although expected to be minimal, the effects of (conditioned) S(+)-ketamine and/or midazolam on QST's for different body locations will be controlled for [33].

4. The change in clinical pain intensity from baseline to directly post-intervention as a result of the pharmacological conditioning with S(+)-ketamine compared to pharmacological conditioning with (combined) placebo medication. Clinical pain intensity is measured with an 11-point numeric rating scale (NRS) ranging from 0 (no pain) to 10 (worst pain imaginable).

5. Extinction of pharmacological conditioning with S(+)-ketamine compared to pharmacological conditioning with (combined) placebo medication is studied by measuring PPT's, wind-up pain, aftersensation, and clinical pain intensity at T = 60mins (directly post-intervention), 75mins, 90mins, 120mins, and 180mins. A significant decrease in difference scores (i.e., a diminishing of the conditioned effect) is considered evidence for extinction [62].

6. The change in Fibromyalgia subjective disease impact from baseline after pharmacological conditioning with S(+)-ketamine, assessed with the Fibromyalgia Impact Questionnaire – Revised (FIQ-R). The FIQ-R consists of 3 domains with a total of 21-items: the physical function domain has 9 items, the

overall impact domain has 2 items and the symptom domain has 10 items. All items are scored on a 11-point numeric rating scale from 0 to 10, with 10 being the worst. The total maximum scores is 100 (scores are summed up per domain and then multiplied by the respective weight of the domain). The questionnaire is has a good internal consistency (Crohnbach's Alpha = 0.95), with item correlations ranging from 0.56-0.93. Unfortunately, test-retest reliability has not been calculated although the original FIQ (1991) had a good reliability (Pearsons' r = 0.56 – 0.95), depending on the domain [132].

7. The presence of adverse effects due to the use of either S(+)-ketamine or midazolam. Psychotomimetic side effects will be assessed with the Bowdle questionnaire (see F.1. Bowdle questionnaire) [133]. The Bowdle questionnaire evaluates three psychedelic ketamine effects, drug high and changes in internal and external perception, from 13 questions scored on a 100-mm VAS scale from zero (no effect) to 100 (maximum effect) [134]. The questionnaire is highly correlated with the concentration of ketamine in the blood and has been studied before in patients with FMS receiving S(+)-ketamine [64, 133]. Nausea and vomiting is assessed as being present or absent (yes or no). Sympathomimetic side effects (tachy or bradycardia, hypo or hypertension, hypoxemia, and hypo or hyperthermia) are studied by measuring the following vital parameters: heart pulse (beats/min), blood oxygen saturation (percentage), systolic and diastolic blood pressure (mmHg), breathing rate (breaths/min), and temperature (degrees Celsius). Monitoring vital parameters is done with a pressure monitoring device and pulse oximeter connected to a bedside monitor, and thermometer (see also F4. SOP studie metingen). Central nervous system side effects (e.g., sedation or agitation) are monitored with the Ramsay Sedation Scale (RSS). The RSS consists of a 6-item scale ranging from 1 (patient is agitated and anxious) to 6 (patient exhibits no response). The overall psychometric properties of the RSS are considered of moderate quality [65].

8. The effects of S(+)-ketamine or midazolam on the variability of respiratory rate and tidal volume as compared to saline. These respiratory parameters are measured continuously from at least 30 minutes prior to infusion of S(+)-ketamine, midazolam or saline, using a non-invasive impedance-based respiratory volume monitor (ExSpiron, Respiratory Motion, Walthan, MA, US, see also D6.4 Manual ExSpiron). Respiratory rate, tidal volume and minute ventilation are recorded as average values over 1minute intervals. In

comparison to spirometry as the gold standard, this monitor is accurate within clinically reasonable limits, with average relative errors for respiratory rate and tidal volume of 1.8% and 9.0% respectively [68]. After baseline measurements prior to infusion of S(+)-ketamine, midazolam or saline, the respiratory measurements will continue during infusion as well as after infusion for at least 120 minutes. Respiratory rate is measured in breaths/minute, changes in tidal volume and minute ventilation are measured as percent of change from baseline. Afterwards, the variability of these parameters is calculated as the coefficient of variation (defined as SD/mean) over intervals of 30 minutes [54].

8.1.3 Other study parameters (if applicable)

Explorative endpoints that will be assessed are demographic, disease-, and trial-related variables: age, sex, disease duration, educational level, body weight, use of escape medication, current use of medicine, accuracy of blinding, and co-morbidities. Presence of severe anxiety or depression is measured with the Hospital Anxiety and Depression Scale (HADS). See also F1.2 Hospital Anxiety and Depression Scale. The HADS contains 14 items that measure anxiety (7-items) and depression (7-tems) symptoms and are scored on a 4-point ordinal scale. The scores for anxiety as well as depression are categorized as: 0-7 normal, 8-10 mild, 11-14 moderate, and 15-21 severe. The HADS is a reliable and valid measure for the assessment of anxiety and depression in chronic pain patients [135, 136]. Additionally, the specificity for predicting a psychiatric disorder is 84% at the cut-off point for severe symptoms, indicating that 16% of people **not** having severe anxiety or depression is at risk of being excluded [137].

8.2 Randomisation, blinding and treatment allocation

Participants will be randomly allocated to one of the three intervention groups: the S(+)-ketamine group, midazolam group or saline group. In the evocation phase all participants will receive saline. The participants are randomized in a 1:1:1 ratio in blocks of variable sizes with CastorEDC. The study intervention is prepared and blinded on the day of testing by the clinical pharmacy of the Amsterdam UMC, location VUmc. Experimenters and participants will be blinded to the allocation of pharmacological intervention.

8.3 Study procedures

Experimenters

The experiment is conducted by the main study experimenter (H.v.L.) and a second assessor. The main experimenter is also a certified medical doctor and is mainly responsible for the well-being of the participant during the experiment. The second assessor will measure QST’s as well as NRS scores.

<u>Recruitment:</u>

Participants will be recruited via outpatient clinics, patient organizations, or with online advertisements via social media (e.g., Facebook). Patients with FMS interested in participation will receive detailed information about the research, particularly on the pressure pain application procedure, the potential side effects of the pharmacological treatments, the hospital visit and discharge, and instructions about food- and drink consumption and driving a car on the day of testing (see E.1. Informatiebrief proefpersonen). Additionally, participants are asked to sign a consent form for pre-experimental screening.

<u>Pre-experimental screening:</u>

Interested patients will be contacted by telephone or e-mail a week in advance of the experiment to make an appointment at the Amsterdam UMC, location VUmc. The main experimenter will verify the pre-experimental consent and ask participants to: 1) fill in an online study screening questionnaire, 2) fill in an online HADS and 3) fill in an electronic pre-operative screening (ePOS). The ePOS is filled out by participants to check the presence of any sedation related risk factors, such as obstructive sleep apnoea, cardiovascular comorbidities, or recreational drug use (see also L6.1 Sedatie PSA – protocol VUmc). The main experimenter will subsequently check eligibility and the results of the ePOS with the study responsible anaesthesiologists to determine if patients can receive the study medication. If the results of the ePOS are not sufficient to decide upon inclusion, patients are contacted by phone to pose any additional questions. Eligible patients are contacted by e-mail to make an appointment at the AUMC and are asked to bring an identity card for a temporary medical record in EPIC. Patients that are ineligible for the study are contacted by e-mail and informed that due to specific study conditions they are not eligible for participation. The main experimenter will randomize eligible patients to the study groups in CastorEDC.

<u>Pre-appointment preparations:</u>

The experiments will be conducted in a recovery room of the anesthesiology outpatient clinics at the Amsterdam UMC, location VUmc. The main experimenter will prepare the study location and check if all necessary materials for the experiment and sedation are present with a checklist (see D6. SOP Medicijntoediening). He will also verify the presence of the on-call anesthesiologist for the study and make sure that the emergency crash car can be found at the right location.

<u>Pre-experimental phase</u>

At the beginning of the appointment in the Amsterdam UMC, patients are asked to create a temporary medical record at the central desk of the outpatient clinics. Next, patients are screened for any COVID-19 related symptoms following the most recent COVID-19 protocols of the Amsterdam UMC (see also). The screening is followed by verbal information about the study procedures and the signing of the informed consent. Specifically, participants are told that they will receive either S(+)-ketamine, a strong painkiller, or placebo medicine and that the dose of the medication is slowly increased to observe how well they tolerate the infusion.

<u>Acquisition phase:</u>

If a participant has provided informed consent and is eligible for study participation, he or she will start with the acquisition phase during the initial visit. The acquisition phase consists of three hospital visits, once every week for three weeks. After the third week, participants are progressing to the evocation phase which consists of one more hospital visit and lasts for one more week (see Figure 1). Every visit in the acquisition and evocation phase consists of the following elements: preparations, baseline measurements, administration of intervention (and measurements), post-intervention measurements, and follow-up (see Figure 3).

<u>Preparations:</u>

Eligible participants will be asked to lay down on a bed in the recovery room to prepare for the baseline measurements. For safety regulations, a time-out procedure is conducted at the beginning of every experiment (see also L6.1 Sedatie PSA – protocol VUmc). The main experimenter will apply a blood pressure monitoring device and pulse oximeter to the dominant arm, and an intravenous catheter to the non-dominant arm. The non-dominant arm is preferred for catheter placement, because the pressure stimuli used for QSTs are applied to the dominant hand. An adhesive electrode is applied to the chest for respiratory measurements and will subsequently be attached to the respiratory volume monitor. Then, the second assessor will explain

the use of the algometer and the QST procedures to the participant and may answer questions if needed. The assessor will also mark the application area of the stimuli (thenar muscles of the dominant hand + ipsilateral tibialis anterior muscle) as part of the QST preparations.

<u>Baseline measurements:</u>

Before the administration of either ketamine, midazolam or saline, the main experimenter will measure blood pressure, arterial haemoglobin-oxygen saturation, heart rate, breathing rate, temperature, and RSS. Participants are excluded if the main experimenter discovers severe hyper or hypotension (diastolic bloodpressure < 50mmHg or > 90mmHg, or systolic bloodpressure < 90mmHg or > 140mmHg), brady or tachycardia (pulse < 50/min or > 100/min), hypoxemia (SpO2 < 94%), hypopnea's (breathing rate < 10/min), or hyperthermia (temp > 37.5 degrees). Respiratory parameters (respiratory rate, changes in tidal volume and minute ventilation) are measured continuously using a non-invasive thoracic electrode starting at least 30 minutes prior to the intervention. After the assessment of these vital parameters (e.g., heart rate), participants are asked to fill in the FIQ-R and Bowdle questionnaire. Next, baseline spontaneous pain and QSTs are assessed by the second assessor. Participants will initially be asked to provide their current clinical pain intensity on an NRS scale ranging from 0 (no pain) to 10 (worst pain imaginable). After verbally rating their spontaneous pain, PPT's are detected according to standardized procedures [127]. The threshold procedure consists of 3 increasing pressure stimuli that are applied to adjacent areas of the thenar muscles and tibialis anterior muscle. In order to optimize force delivery and minimize skin irritation, the algometer probe (1cm2) is held perpendicular to the muscle belly. The pressure is increased with 1kg/s until participants report their first sensation of pain, which is also scored on an NRS scale. After a 2 min. interval, assessment of differences in temporal summation are investigated by applying a sequence of 10 pressure stimuli at threshold level to the same muscle groups [1, 2, 15]. Every stimulus consists of a 2 second pressure increase (rate 2kg/s) and a 1 second plateau phase where maximum pressure is held constant. The interval between every stimulus (ISI) is 1 second and stimuli are thus applied every ~ 3 seconds (frequency ~ 0.33Hz). The specific frequency is the most applied for temporal summation assessments and has to do with the firing rate of the unmyelinated C-fibers responsible for the initiation of windup [14, 15, 138]. After a single sequence of pressure stimuli, participants are asked to rate their late painful sensations after 15 seconds on an NRS scale to assess aftersensation (see Fig. 4) [139].

Administration of intervention:

When all baseline measurements have been conducted, participants are attached to the IV pumps and instructed about the start of the study medication. The main study experimenter will inform participants that they will receive their allocated study medication, which can be either a strong painkiller, or a placebo, over the next hour. The intravenous infusion of the allocated intervention is then started for one hour. In the acquisition phase, participants will receive either the S(+)-ketamine, midazolam or saline treatment unbeknownst to the experimenters. The main experimenter will increase the pump flow according to the step-up dosing paradigm described in section 6.5. During the one hour administration, participants' vital signs will be measured every 5 minutes (12 times in total). Respiratory parameters will be measured continuously throughout the intervention. Also, clinical pain, QSTs, the RSS, the Bowdle questionnaire and nausea and vomiting will be assessed after 30 and 60 minutes. The Modified Early Warning Score (MEWS) will be used for standardized evaluation of the participant's vital functions in accordance with the VUmc patient safety protocols (see D6. SOP Medicijntoediening). The administration is halted when: 1) participants' MEWS score exceeds 3, 2) severe side effects are occurring, or 3) participants indicate that they can no longer tolerate the side effects. The on-call anesthesiologist is contacted via phone (sein *986151) when side effects occur that need attention of a senior medical doctor (e.g., for the prescription of anti-emetic medication). In the case of serious psychiatric adverse events, the psychiatric consultative service (PCD) of the Amsterdam UMC can be contacted via phone (sein* 61491). Finally, an unimproved MEWS score for more than 1 hour or the existence of persisting apnea's will lead to communication with the Spoed Interventie Team (SIT) from the Amsterdam UMC, location VUmc (sein *61275) about the situation and a possible co-evaluation of the participants' health status.

Post-intervention testing:

The infusion of medication will be stopped after an hour of administration. Participants are immediately detached from the infusion pumps. In the 2 hours after the intervention has stopped participants are asked to remain in bed in the recovery room. Monitoring of vital parameters is continued for every 15 minutes. Clinical pain intensity, QSTs and the RSS are assessed at 75, 90, 120 and 180 minutes after the start of the infusion and at the same time participants will be asked to fill in the Bowdle questionnaire. In between measurements, participants are provided with food and water. Continuous respiratory volume monitoring will continue for at least 2 hours

after stopping administration of medication. After the 2 hour follow-up, the main experimenter will check if participants are able to leave the hospital. A participant is ready to leave the hospital if he/she meets all criteria for discharge as stated in the sedation protocol of the VUmc (see also L6.1 Sedatie PSA – protocol VUmc). In case the main experimenter is uncertain about the physical or mental health of a participant, he will first contact the study involved anaesthesiologist and if necessary also contact the psychiatric consultative services of the AUMC. Participants that are unable to leave the hospital will be monitored until they fulfil the discharge criteria. Participants are also instructed to arrange a familymember or friend that can escort them back home after the experiment to ensure safety upon discharge.

<u>Evocation phase:</u>

The preparations and baseline measurements in the evocation phase and acquisition phase are equal. Similar to the acquisition phase, participants are attached to a continuous IV pump. The difference between the acquisition and evocation phase is that in the evocation phase all participants will receive IV saline. However, similar to the acquisition phase, participants are instructed that they will receive their allocated study medication. The verbal instructions of the acquisition and evocation phase are equal to optimally elicit a conditioned response. The infusion procedure follows the same timeline as in the acquisition phase. Vital parameters and RSS will be closely monitored and after an hour the infusion is stopped by the main experimenter. Continuous respiratory monitoring is performed before, during and after the evocation phase, similar to the acquisition phase. The outcomes are measured by the second assessor at baseline, during infusion (at 30 and 60 minutes), and post-infusion (at 75, 90, 120, and 180 minutes), similar to the acquisition phase.

<u>Follow-up:</u>

Upon finishing the appointment, participants will receive the FIQ-R, which they are asked to fill in after one week. After the final appointment, participants are also contacted by phone after one week to assess their well-being and discover any adverse events that occurred during the follow-up.

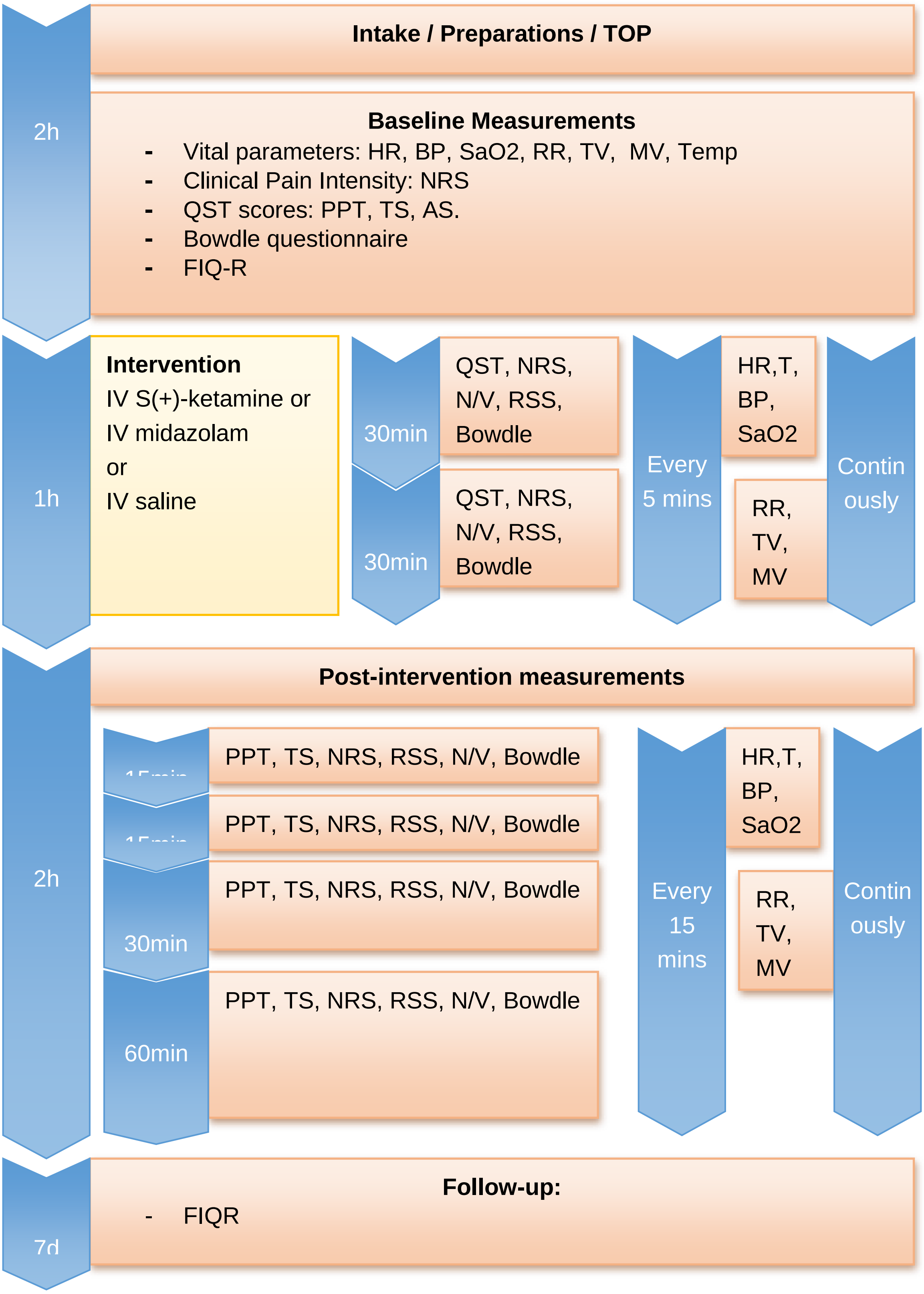

Fig. 3: Procedure of hospital visit: HR: heart rate, BP: blood-pressure, ePOS: electronic preoperative screening, SaO2: arterial oxygen saturation, RR: respiratory rate, TV: tidal volume, MV: minute ventilation, NRS: numeric rating scale, PPT: pressure pain threshold, RSS: Ramsay Sedation Scale, Temp: body temperature, TOP: time-out procedure, TS: temporal summation, VP: vital parameters, N/V: nausea & vomiting.

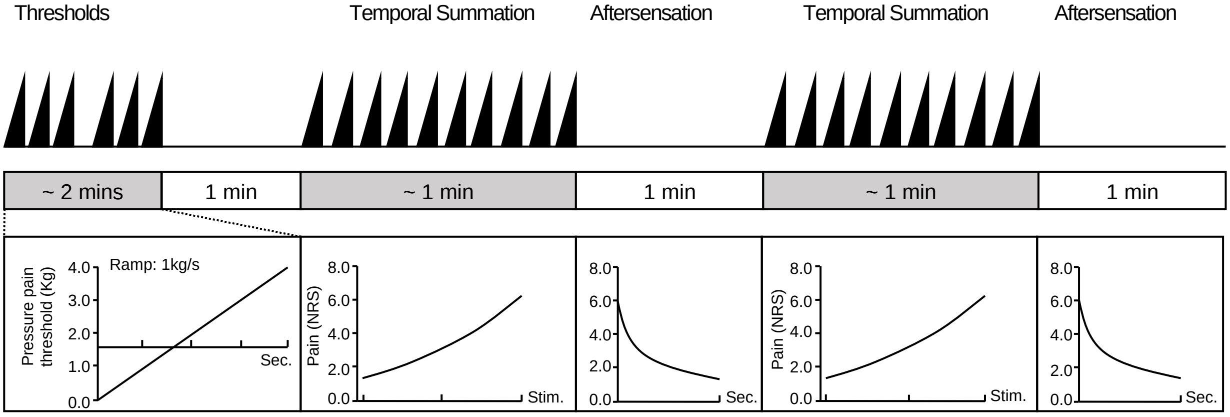

Fig. 4: overview of pressure QST's. Thresholds are determined by slowly increasing the pressure on three different locations of the respective muscles (thenar and tibialis anterior) after which the forces are averaged for every muscle. Temporal summation is measured by scoring the pressure pain every 1st, 5th, and 10th stimulus, after which the aftersensation pain is measured at 15 seconds. A pause of 1 min. between threshold testing and temporal summation is necessary to prevent peripheral sensitization of the muscle tissue. NRS: numeric rating scale, Stim.: stimulus

8.4 Withdrawal of individual subjects

Subjects can leave the study at any time for any reason if they wish to do so without any consequences. The investigator can decide to withdraw a subject from the study for urgent medical reasons.

8.4.1 Specific criteria for withdrawal (if applicable)

Not applicable

8.5 Replacement of individual subjects after withdrawal

Participants who voluntarily withdraw from the study prematurely, or who are withdrawn at the discretion of the researcher for technical or medical reasons will not be replaced. All data from participants that asked to be withdrawn from the study will be excluded from analysis.

8.6 Follow-up of subjects withdrawn from treatment

Participants who withdraw from the study prematurely for medical reasons will receive at least one follow-up contact by telephone soon after withdrawal. Additional follow-up contacts will be made if necessary until the participant has recovered completely.

8.7 Premature termination of the study

Not applicable

9. SAFETY REPORTING

9.1 Temporary halt for reasons of subject safety

In accordance to section 10, subsection 4, of the WMO, the sponsor will suspend the study if there is sufficient ground that continuation of the study will jeopardise subject health or safety. The sponsor will notify the accredited METC without undue delay of a temporary halt including the reason for such an action. The study will be suspended pending a further positive decision by the accredited METC. The investigator will take care that all subjects are kept informed.

9.2 AEs, SAEs and SUSARs

9.2.1 Adverse events (AEs)

Adverse events are defined as any undesirable experience occurring to a subject during the study, whether or not considered related to the investigational product. All adverse events reported spontaneously by the subject or observed by the investigator or his staff will be recorded.

9.2.2 Serious adverse events (SAEs)

A serious adverse event is any untoward medical occurrence or effect that

- results in death;
- is life threatening (at the time of the event);
- requires hospitalisation or prolongation of existing inpatients' hospitalisation;
- results in persistent or significant disability or incapacity;
- is a congenital anomaly or birth defect; or
- any other important medical event that did not result in any of the outcomes listed above due to medical or surgical intervention but could have been based upon appropriate judgement by the investigator.

An elective hospital admission will not be considered as a serious adverse event.

Previous studies have used the same or a higher dose of ketamine or midazolam without any serious adverse events occurring [64, 83, 95, 140, 141]. As such, we do not expect any SAEs to occur. On the rare occasion that they do occur, however, the investigator will report all SAEs through the web portal ToetsingOnline to the accredited METC that approved the protocol, within 7 days of first knowledge for SAEs that result in death or are life threatening, followed by a period of maximally 8 days to complete the initial preliminary report. All other SAEs will be reported within a period of maximally 15 days after the sponsor has first knowledge of the serious adverse events.

9.2.3 Suspected unexpected serious adverse reactions (SUSARs)

Adverse reactions are all untoward and unintended responses to an investigational product related to any dose administered.

Unexpected adverse reactions are SUSARs if the following three conditions are met:

1. the event must be serious (see chapter 9.2.2);
2. there must be a certain degree of probability that the event is a harmful and an undesirable reaction to the medicinal product under investigation, regardless of the administered dose;
3. the adverse reaction must be unexpected, that is to say, the nature and severity of the adverse reaction are not in agreement with the product information as recorded in:
   - Summary of Product Characteristics (SPC) for an authorised medicinal product;

- Investigator's Brochure for an unauthorised medicinal product.

The sponsor will report expedited the following SUSARs through the web portal *ToetsingOnline* to the METC

- SUSARs that have arisen in the clinical trial that was assessed by the METC;
- SUSARs that have arisen in other clinical trials of the same sponsor and with the same medicinal product, and that could have consequences for the safety of the subjects involved in the clinical trial that was assessed by the METC.

The remaining SUSARs are recorded in an overview list (line-listing) that will be submitted once every half year to the METC. This line-listing provides an overview of all SUSARs from the study medicine, accompanied by a brief report highlighting the main points of concern.
The expedited reporting of SUSARs through the web portal Eudravigilance or ToetsingOnline is sufficient as notification to the competent authority.

The sponsor will report expedited all SUSARs to the competent authorities in other Member States, according to the requirements of the Member States.

The expedited reporting will occur not later than 15 days after the sponsor has first knowledge of the adverse reactions. For fatal or life threatening cases the term will be maximal 7 days for a preliminary report with another 8 days for completion of the report.

9.3 Annual safety report

In addition to the expedited reporting of SUSARs, the sponsor will submit, once a year throughout the clinical trial, a safety report to the accredited METC, competent authority, and competent authorities of the concerned Member States.
This safety report consists of:

- a list of all suspected (unexpected or expected) serious adverse reactions, along with an aggregated summary table of all reported serious adverse reactions, ordered by organ system, per study;
- a report concerning the safety of the subjects, consisting of a complete safety analysis and an evaluation of the balance between the efficacy and the harmfulness of the medicine under investigation.

9.4 Follow-up of adverse events

All AEs will be followed until they have abated, or until a stable situation has been reached. Depending on the event, follow-up may require additional tests or medical procedures as indicated, and/or referral to the general physician or a medical specialist. SAEs need to be reported till end of study within the Netherlands, as defined in the protocol.

9.5 [Data Safety Monitoring Board (DSMB) / Safety Committee]

A DSMB will not be utilized in this study. This decision has been made according to the guideline from the Nederlandse Federatie van Universitaire Medische Centra; kwaliteitsborging mensgebonden onderzoek 2019. Although an intermediate risk for damage is anticipated in this study (see also K6. Risicoclassificatie V07 – F01). The predominant reason for this intermediate risk is the occurrence of side effects due to study drug administration. As multiple safe guards have been implemented to either prevent or lessen side effects (e.g., low drug dose, step-up dose regimen, and preoperative screening and time-out procedure), a DSMB seems less necessary for this study.

10. STATISTICAL ANALYSIS

Descriptive statistics of relevant variables will be calculated. Continuous outcomes will be shown by means and standard deviations, dichotomous outcomes by numbers and percentages. The main statistical analysis used in this study is a linear mixed model (LMM) conducted with Rstudio [142]. Assumptions of all statistical analyses will be checked by visually inspecting raw data and appropriate measures will be taken in case of violation (e.g., transformation, nonparametric testing, or bootstrapping). Tests will be performed two-sided with an alpha level of $p \leq 0.025$ to determine statistical significance of the results. This alpha level is corrected for two multiple comparisons (three fixed factors) according to the Bonferroni correction [143]. Effect sizes will be calculated for each effect (e.g., Cohen's f^2). Multilevel regression analysis, used as the main statistical method for analyzing the results, is to a lesser extent affected by drop-outs and thus favourable for our proof-of-principle study [144].

10.1 Primary study parameter(s)

The primary analysis used to study the influence of pharmacological conditioning with S(+)-ketamine compared to pharmacological conditioning with placebo medication in the evocation phase is going to be executed with an LMM model. The analysis will follow the intention-to-treat principle and include all participants that finished the infusion of the

evocation phase, regardless of the total drug dose received. An additional per-protocol analysis shall be conducted to gain further insight into the effect of optimal dosing on pharmacological conditioning. The main parameter for the primary analysis is the difference in PPT levels between baseline and directly post-intervention (at T = 60mins) averaged out over two body locations (hand and lower limb). The primary LMM model is constructed from three fixed factors (group, time, and the interaction term of group x time) and one random factor (participants). Inferential testing for the regression analysis is executed by bootstrapping the results. A within-subject analysis is initially conducted to assess the conditioned effects for each specific group separately (S-ketamine, midazolam, or saline). After the within-subject analysis, between subject analyses are conducted to test for differences between groups in the evocation phase.

10.2 Secondary study parameter(s)

The secondary parameters conditioned effects of S(+)-ketamine on PPT's, aftersensations, clinical pain intensity and subjective disease impact are also going to be analysed with the same LMM model as described above. The secondary analyses will also follow the intention-to-treat principle. A within-subject analysis is initially conducted to investigate the conditioned effects for wind-up and aftersensations, clinical pain and the disease impact. After the within-subject analysis, between-subject analyses will be conducted for the evocation phase. The influence of body location on pharmacological conditioning is studied by using the same LMM model with an additional fixed factor for body location. Extinction of conditioned effects is examined by expanding the time factor of the primary LMM model. The influence of pharmacological conditioning on psychotomimetic and sympathicomimetic side effects is studied with a within-subject analysis for every group. With regard to the variability of breathing, this will be analysed with an LMM model as described before. In the within-subject analysis, we will investigate the effects of the intervention over time. A between-subject analysis will be performed to test for differences between treatment groups.

Inferential testing is again executed by bootstrapping the results.

10.3 Other study parameters

The exploratory parameters will be inspected and shown with descriptive statistics. Further analysis of possible relationships between exploratory parameters and study outcomes is done by treating them as either covariates, or moderators, or mediators depending on the specific variable and/or assumptions that need to be met. The following parameters are explored as possible covariates or moderators (depending on the

assumptions met): age, sex, disease duration, educational level, body weight, comorbidities, current use of medication. The use of escape medication is explored as a possible mediator. Any exploratory variable can be analysed as covariate, moderator or mediator by adjusting the regression analysis of the previously described LMM models. Inferential statistics are then calculated by bootstrapping the results [145].

10.4 Interim analysis (if applicable)

Not applicable

11. ETHICAL CONSIDERATIONS

11.1 Regulation statement

The study will be conducted according to the principles of the Declaration of Helsinki (2 October, 2013), in accordance with the Medical Research Involving Human Subjects Act (WMO) and the General Data Protection Regulation (GDPR, also known as AVG). The protocol of this study will be submitted to the “Medisch Ethische Toetsings Commissie” (METC), Leiden-Den Haag-Delft, and the study will not commence before formal approval has been granted. All researchers involved in these projects are BROK (Basiscursus Regelgeving en Organisatie voor Klinisch onderzoekers) certified.

11.2 Recruitment and consent

Adult female patients with FMS will be recruited via patient organizations, social media, and treatment centers in the Netherlands. If a person indicates interest in participating, she will be provided with information about the study in writing and asked to fill in a pre-consent form, as well as an pre-experimental screening questionnaire. Next, she will be invited to the Amsterdam UMC, location VUmc, where additional questions or information can be clarified by the main experimenter. Afterwards, she will be asked to sign the in-formed consent form and screened for eligibility.

Objection by minors or incapacitated subjects (if applicable)

Not applicable

11.3 Benefits and risks assessment, group relatedness

Although the current study is predominantly a proof-of-principle study, patients with FMS might benefit from participation depending on the allocated group. Previous studies have shown an analgesic effect of IV ketamine administration in patients with FMS. However, the duration of analgesia differs across studies [31, 33, 64, 146]. Participants allocated to

the S(+)-ketamine group might therefore experience short-lived relief of their pain symptoms. Patients with FMS in the IV midazolam or IV saline groups are not expected to benefit from their allocated intervention, except for any experienced placebo analgesic effects. Some researchers pose that benzodiazepines are beneficial for pain sensitization but the strength of the evidence supporting this statement is low [78-82]. Risks in the current trial are expected to be minimal. Ketamine (or it's S(+)-enantiomer) has been administered to patients with FMS in similar trials with higher doses, without the occurrence of serious adverse events [31, 33, 64]. The most reported side effects in these trials were: psychotomimetic effects (hallucinations or dissociations), dysphoria, nausea, vomiting, or headaches. Additionally, the implementation of a step-up-dose regimen will help to ameliorate any disturbing side effects and reduce the possibility of any serious adverse events to occur. The major side effects of midazolam are cardiovascular or respiratory and become prominent at higher doses than used in this study (max. 0.05mg/kg/h). In a study investigating continuous infusion of IV midazolam with a dosage of ~0.13mg/kg/h in patients with FMS, apneas, hypoxemia, or hypotension was not observed [64]. Overall few serious adverse effects due to the administration of research medication is expected, predominantly sleepiness, psychotomimetic effects, dissociation, and nausea are anticipated due to IV S(+)-ketamine or IV midazolam. In the unlikely event that a serious side effect will occur, the study contains multiple safeguards to facilitate a rapid and accurate treatment of participants.

The handheld analogue algometer (Force Dial, Wagner Instruments, see also D6.3 Manual Algometer) has been successfully used in many research laboratories, including the LUMC, over many years, without producing any injury. The pressure that elicits pain will be chosen on an individual level for each participant, thus, participants will not receive a stimulation that is unbearable for them. Previous studies with a handheld algometer by Wagner instruments have also been executed in patients with FMS without the occurrence of injury [1, 2, 147]. Nevertheless, mild discomfort or anxiety may be experienced during the safe and standardized inductions of pain.

The respiratory volume monitor (ExSpiron, Respiratory Motion, Walthan, see also D6.4 Manual ExSpiron) is frequently used in clinical practice to measure and trend respiratory rate and tidal volume in non-intubated patients. It consists of a non-invasive, adhesive, thoracic electrode connected to a monitor. There is no discomfort or risk aside from wearing an adhesive sticker. The measurements are performed continuously without additional stimulus to the patient.

11.4 Compensation for injury

The investigator has a liability insurance in accordance with article 7 of the WMO. The sponsor (also) has an insurance which is in accordance with the legal requirements in the Netherlands (Article 7 WMO). This insurance provides cover for damage to research subjects through injury or death caused by the study. The insurance applies to the damage that becomes apparent during the study or within 4 years after the end of the study. For details regarding liability insurances, please refer to appendices G1 and G2.

11.5 Incentives (if applicable)

Participants will receive a reimbursement of €120,- for completion of the study. The reimbursement is offered because the aim of the study is primarily experimental (non-therapeutical) and participants will have to invest a lot of time and effort into completing this study. Participants are therefore payed according to the CCMO guidelines following the wage payment model. Considering the minimum wage in the Netherlands to be ~ €6/hour (see also: https://www.rijksoverheid.nl/onderwerpen/minimumloon/bedragen-minimumloon/bedragen-minimumloon-2020) a participation in the entire experiment should be reimbursed as; 4 visits * 5 hours * €6 = €120,-. See also J1 Informatie vergoeding proefpersonen.

12. ADMINISTRATIVE ASPECTS, MONITORING AND PUBLICATION

12.1 Handling and storage of data and documents

CastorEDC is utilized to create pseudonomyzed electronic case report forms. Participant identification codes will be used to link data to participants. The file containing the linking between participant's number and personal data (e.g., name) will be managed by the researchers and data manager and will be locked for access by others. Collected data (e.g., questionnaires, informed consent, NRS values) will be stored for a period of at least 15 years. Best practices will be followed as recommended in the Code of Conduct for the Use of Data in Health Research (Commissie Regelgeving en Onderzoek) and theGeneral Data Protection Regulation (GDPR).

12.2 Monitoring and Quality Assurance

Monitoring for the conduct of the study will be done by the Data Manager of the Health, Medical and Neuropsychology (HMN) unit of Leiden University, who will check whether the data that have been entered in the database matches the source data by random sampling. As per the protocol that has been set up for data management in the HMN unit, the data manager will be responsible for the initiation visit, initial monitoring after first 3 participants, half-yearly checks and the final check of the data at the end of the study. We kindly refer to the document “”K6.3 Monitorplan KetCOP”.

12.3 Amendments

Amendments are changes made to the research after a favourable opinion by the accredited METC has been given. All amendments will be notified to the METC that gave a favourable opinion.

A ‘substantial amendment’ is defined as an amendment to the terms of the METC application, or to the protocol or any other supporting documentation, that is likely to affect to a significant degree:

- the safety or physical or mental integrity of the subjects of the trial;
- the scientific value of the trial;
- the conduct or management of the trial; or
- the quality or safety of any intervention used in the trial.

All substantial amendments will be notified to the METC and to the competent authority.

Non-substantial amendments will not be notified to the accredited METC and the competent authority, but will be recorded and filed by the sponsor.

12.4 Annual progress report

The sponsor/investigator will submit a summary of the progress of the trial to the accredited METC once a year. Information will be provided on the date of inclusion of the first subject, numbers of subjects included and numbers of subjects that have completed the trial, serious adverse events/ serious adverse reactions, other problems, and amendments.

12.5 Temporary halt and (prematurely) end of study report

The sponsor will notify the accredited METC and the competent authority of the end of the study within a period of 90 days. The end of the study is defined as the last patient’s last visit.

The sponsor will notify the METC immediately of a temporary halt of the study, including the reason of such an action.

In case the study is ended prematurely, the sponsor will notify the accredited METC and the competent authority within 15 days, including the reasons for the premature termination.

Within one year after the end of the study, the investigator/sponsor will submit a final

study report with the results of the study, including any publications/abstracts of the study, to the accredited METC and the Competent Authority.

12.6 Public disclosure and publication policy

In accordance with the CCMO statement on publication policy, the results of this study will be disclosed unreservedly, i.e. regardless of confirmation or disconfirmation of the hypotheses. The results will be submitted for publication in peer-reviewed journals.

13. STRUCTURED RISK ANALYSIS

13.1 Potential issues of concern

a. Level of knowledge about mechanism of action

S(+)-Ketamine:

Esketamine, the (S+)-enantiomer of racemic ketamine, belongs to the pharmacotherapeutic group of anesthetics and to the class of organic compounds known as chlorobenzenes. Ketamine hydrochloride is a cyclohexanone derivative used for induction of anesthesia. The general anesthetic ketamine is known to be a selective NMDA receptor antagonist. It binds specifically to the dizocilpine (MK-801) site of the NMDA receptor, near the channel pore and is an uncompetitive antagonist. Ketamine is most often administered via a continuous IV pump-infusion for 100% bioavailability. Ketamine is absorbable by an intravenous route due to both its water and lipid solubility and excretion is mainly renal. Peak plasma concentrations after IV administration are reached within 2 to 10 minutes. See also in SPC (‘D2. SPC Ketanest’) and product information (‘D2.2 Ketanest Bijsluiter’).

Midazolam:

Midazolam is a pharmaceutical agent that belongs to the class of imidazobenzodiazepines and also a member of monofluorobenzenes. It is commonly used for sedation and hypnosis. Benzodiazepines exert their inhibitory effects by potentiating the effect of GABA on the GABA-A receptor. The allosteric action of benzodiazepines on the GABA-A subunit of the receptor causes an increased sensitivity of the GABA-receptor for GABA itself. Resultantly, the activated GABA receptor opens a nearby chloride channel facilitating hyperpolarization, which leads to depression of the central nervous system [148]. Midazolam is, like ketamine, often administered via a continuous IV pump-infusion for 100% bioavailability. Midazolam is readily absorbable by an intravenous route due to its

water solubility, mainly glucuronidated by the liver and predominantly excreted via the kidneys. Peak plasma concentration after IV administration is reached within seconds and correlates with the occurrence of clinical symptoms [87]. See also in SPC (‘D2. SPC Midazolam Aurobindo’) and product information (‘D2.2 Midazolam Bijsluiter’).

<u>Sodium Chloride 0,9% (saline)</u>

Sodium chloride 0.9% belongs to the crystalloid fluids that consist of water solutions with small inorganic ions and small organic molecules [88]. It is commonly used for fluid-depleted patients, dissolving of medication for intravenous application, or cleansing of medical materials [88]. Excretion of sodium and chloride is fully renal. See also in SPC (‘D2. SPC Natriumchloride’) and product information (‘D2.2 Natriumchloride Bijsluiter’)

<u>b. Previous exposure of human beings with the test product(s) and/or products with a similar biological mechanism</u>

<u>S(+)-Ketamine:</u>

In various recent experimental studies, comparable or higher doses of ketamine have been administered intravenously to human participants and have led to significant analgesic effects [64, 84, 86, 95, 149, 150]. Dosages often have exceeded the dose used in this study [64, 74, 95, 97], with the higher sub-anesthetic dosages of 0.4mg/kg not associated to side effects [76, 97, 98]. Additionally, a step-up dose regimen of S(+)-ketamine was successfully implemented in a study with healthy participants and the results showed that analgesia was achieved with similar dosages to the current study [96]. Intravenous S(+)-ketamine has also been administered frequently to patients with FMS in comparable doses [31, 33, 64]. Therefore, a step-up dose regimen of 0.1 – 0.2 – 0.3mg/kg/h of IV S(+)-ketamine is an effective and safe dose.

<u>Midazolam:</u>

Midazolam has been administered via an IV route in other clinical trials utilizing equal or higher doses without the occurrence of severe side effects [106-108, 151]. A comparable study with patients with FMS administered 5mg of Midazolam in 0.5 hours in patients averaging 77 kilograms, which is equal to 0.13mg/kg/h [64]. In this study, oxygen saturations remained well above 98%, depicting good clinical safety of the doses utilized in this study (0.017 – 0.033 – 0.05mg/kg/h) [64].

<u>Sodium chloride 0,9% (saline):</u>

Sodium chloride has been administered as a placebo intravenous intervention in many trials [59, 76, 83-85]. Side effects are only observed when participants receive large amounts of sodiumchloride (2 liter in 1 hour ~ 30ml/kg/h), which is not comparable to our study (50ml in 1 hour ~ 0.7ml/kg/h) [111].

c. Can the primary or secondary mechanism be induced in animals and/or in *ex-vivo* human cell material?

Not applicable

d. Selectivity of the mechanism to target tissue in animals and/or human beings

S(+)-Ketamine:

We kindly refer to the Summary of Product Characteristics (‘D2. SPC Ketanest’) and product information (‘D2.2 Ketanest Bijsluiter’) for information about the selectivity of the mechanisms of S(+)-ketamine.

Midazolam:

We kindly refer to the Summary of Product Characteristics (‘D2. SPC Midazolam Aurobindo’) and product information (‘D2.2 Midazolam Aurobindo Bijsluiter’) for information about the selectivity of the mechanisms of midazolam.

Sodium Chloride 0.9% (saline):

We kindly refer to the Summary of Product Characteristics (‘D2. SPC Natriumchloride’) and product information (‘D2.2 Natriumchloride Bijsluiter’) for information about the selectivity of the mechanisms of sodium chloride.

e. Analysis of potential effect

S(+)-ketamine:

In the current study one third of the participants will receive a step-up dose regimen of 0.1 – 0.2 – 0.3mg/kg/h S(+)-ketamine in 1 hour. The expected analgesic effect of this proposed regimen is partially based upon the findings a previous study that used a comparable step-up dose regimen (0.05 – 0.1 – 0.15mg/kg/h) for 1.5 hours showing significant analgesia [96]. Although the efficacy of this regimen seems promising, we use a somewhat higher dose in the current study, because other studies showed that a higher minimum dose of 0.3mg/kg/h or 15mg/70kg/h of S(+)-ketamine was required to achieve analgesia [74, 86]. Side effects reported in studies utilizing continuous subanesthetic IV

S(+)-ketamine infusions were nausea and vomiting, dizziness, sedation, or hallucinations. These studies used dosages ranging from 0.1mg to 0.57mg per kg [75, 86, 95, 97, 98]. Similar side effects were found in studies that investigated ketamine's analgesic effect in patients with FMS [31, 33, 64]. Due to the fact that the dose administrated in the current study is lower than the dosages administered in the previous studies (max. 0.3mg/kg/h), no serious adverse effects are expected. Patients with FMS may however be more susceptible to some of the psychotomimetic side effects, including dysphoria , which already can occur at S(+)-ketamine concentration <100ng/ml, corresponding to a dosis of 0.3mg/kg/h for one hour. [86]. We have therefore decided to implement the step-up dose regimen as an extra safeguard. By gradually increasing the dosage, patients can accommodate more gradually to any potential symptoms and are able to stop at any time when they experience too many symptoms, which will then improve more quickly than when a full dose would have been provided from the start. Resultantly, with all of the previous evidence in mind, the current step-up dose regimen is considered both effective in providing pain relief and safe.

<u>Midazolam:</u>

Midazolam is administered to one third of the participants in this study and will be used as an active placebo because of its sedative properties. The IV administration of midazolam runs in a similar fashion to the S(+)-ketamine administration with a step-up dose regimen of 0.017 – 0.033 – 0.05mg/kg/h, which equals to a total dose of 0,39mg – 0,76mg – 1,16mg for a person of 70kg. A sedative dose of midazolam is between 2 and 7.5mg i.v., which means that the total dose of the current regimen could effectively induce a sedative state [87]. The most reported side effects of midazolam are due to a depression of the central nervous system (sleepiness, drowsiness, confusion or blurred vision), the cardiovascular system, and/or respiratory system [99]. Cardiovascular and respiratory side effects (hypotension, hypoxemia, or apnea) are observed when an anesthetic bolus dose (0.15mg/kg) or maintenance dose (0.1 - 0.3mg/kg/h) is administered [101]. Studies investigating conscious sedation with midazolam showed no apnea or hypoxemia when doses of ≤0.07mg/kg were given [102-105]. In a similar study with patients with FMS, no adverse effects occurred when a dose of 5mg midazolam was administered in 0.5 hour (~ 0.13mg/kg/h if corrected for the average weight) [64]. The step-up dose regimen of 0.017 – 0.033 – 0.05mg/kg utilized in this study is thus considered safe and effective.

<u>Sodiumchloride 0,9% (saline):</u>

As a consequence of its composition, the most frequent reported side effects are hyperchloremia, metabolic acidosis, and renal failure [88]. However, these side effects are

observed when participants receive large amounts of saline (2 liter in 1 hour ~ 30ml/kg/h) [111]. In this study a maximum of 50ml saline is infused, so risks of metabolic changes are considered nil.

f. Pharmacokinetic considerations

Ketamine:

See the Summary of Product Characteristics (‘D2. SPC Ketanest’) and product information (‘D2.2 Ketanest Bijsluiter’).

Midazolam:

See the Summary of Product Characteristics (‘D2. SPC Midazolam Aurobindo’) and product information (‘D2.2 Midazolam Aurobindo Bijsluiter’).

Sodium Chloride 0.9% (saline):

See the Summary of Product Characteristics (‘D2. SPC Natriumchloride’) and product information (‘D2.2 Natriumchloride Bijsluiter’).

g. Study population

In this study, female patients suffering from Fibromyalgia syndrome are included. Fibromyalgia syndrome is a chronic widespread pain condition. Patients often experience pain hypersensitivity in the muscular area of limbs. Possible patients who are pregnant, have a severe co-morbidity, are allergic to the study medication, or have to use medication that interacts with the study medication will be excluded.

h. Interaction with other products

A systematic assessment of interactions for the investigational medicinal products and other medication was carried out. The Dutch governmental website of pharmaceutical medicine (www.farmaceutischkompas.nl) was used to investigate drug interactions. Saline is not investigated due to its chemical properties.

S(+)-Ketamine:

The parasympathomimetic cardiovascular effects of S(+)-ketamine might be increased when administered simultaneously with parasympathomimetics (bronchodilators), thyroid hormones or vasopressin. The sedative effects are increased with concomitant use of opioids, and S(+)-ketamine can also further depress the central nervous system effects of opioids. Medicine that inhibit the CYP3A4 enzymes (e.g., verapamil, diltiazem or certain antibiotics) can decrease liver metabolization, which requires a lowering in dose. On the other hand, medicine that induce CYP3A4 enzymes (e.g., rifampicin, carbamazepine,

fenytoin) need to be accounted for with a higher dose. Important to note is TCA's and selective noradrenaline re-uptake inhibitors (SNRIs) have sympathomimetic effects that can interact with S-ketamine's cardiovascular effects (hypertension). Patients utilizing medicine that inhibit or stimulate CYP3A4 enzymes and/or use strong opioids will be excluded. Patients with FMS that use TCA's, SNRI's or weak opioids for their pain will be closely monitored and will be asked to keep their medication consumption at a constant level.

Midazolam:

The centrally inhibiting effects of opioids and benzodiazepines can act synergistic when administrated simultaneously. Patients utilizing strong opioids will therefore be excluded. Interactions with CYP3A4 influencing medicine is mostly applicable for oral administration; however, they might also affect intravenous administration. Patients utilizing TCAs, other benzodiazepines and tramadol will therefore be closely monitored. In addition, patients will be asked to keep their medication consumption at a constant level throughout the study.

i. Predictability of effect

Not applicable

j. Can effects be managed?

S(+)-Ketamine: See SPC ('D2 SPC Ketanest') and product information ('D2.2 Bijsluiter Ketanest'). Access to adequate medical support in case of emergencies is arranged via emergency support for the Amsterdam UMC, location VUmc, an on-call physician at the Amsterdam UMC, the psychiatric consultative services (PCD), and other medical staff involved in this research. There are no specific antidotes for S-ketamine, benzodiazepines can be administered to lower psychotomimetic side effects.

Midazolam: See SPC ('D2 SPC Midazolam Aurobindo') and product information ('D2.2 Bijsluiter Midazolam Aurobindo'). Midazolam can be antagonized with flumazenil, which is a short-acting (2-3 hours) benzodiazepine antagonist [148]. Flumazenil is usually administered intravenously with a loading dose of 0.2mg and can be titrated up to a maximum of 1mg with increments of 0.1mg per 60 seconds [87].

Sodiumchloride 0,9% (saline):

See SPC ('D2. SPC Sodiumchloride') and product information ('D2.2 Bijsluiter Sodiumchloride'). In case sodium or chloride blood levels are very high, infusion with

water can be administered to lower the concentrations. However, this situation is not likely to happen in the study due to the very low amount of Sodiumchloride administered (50ml = 15,4mmol sodium and 15.4 mmol chloride).

13.2 Synthesis

S(+)-Ketamine:

The risks associated with the administration of S(+)-ketamine at sub-anesthetic doses of 0.1 – 0.2 – 0.3mg/kg/h in our study are minimal [64, 86]. Participants will be extensively and carefully screened during the standard electronic pre-operative screening (ePOS) for factors that may increase chances of unwanted side effects, such as specific severe co-morbidities or severe psychiatric conditions and the use of co-medication. Patients will be especially assessed for the presence of severe cardiovascular disease (angina), hyperthyroidism or thyroid medication use, increased intraocular pressure or liquor obstructive states [93]. Patients well-being is also continuously monitored throughout the study and S(+)-ketamine is administered in increments providing early detection of any non-tolerable or serious side effects. Access to adequate medical support in case of emergencies is arranged via emergency support and an on-call anesthesiologist at the Amsterdam UMC, the psychiatric consultative services (PCD), and other medical staff involved in this research. Participants who report any side effects will be monitored at the Amsterdam UMC until it is determined that it is safe for them to depart.

Midazolam:

There are minimal risks associated with the administration of sub-anesthetic doses of midazolam at a dose of 0.017 – 0.033 – 0.05mg/kg/hour in this study [103]. Patients will be continuously monitored throughout the study and the step-up dose regimen provides early detection of any non-tolerable or serious side effects (e.g., apneas or hypoxemia). In case of respiratory depression (prolonged apneas > 10 seconds, or hypoxemia <94%), the experimenter will try to arouse the participant and abolish testing, then call for help by contacting the on-call anesthesiologist of the Amsterdam UMC to decide on administration of flumazenil and possible further actions. Participants who report any side effects will be monitored until it is determined that it is safe for them to depart.

14. REFERENCES

1. Coppieters, I., et al., *Effects of Stress and Relaxation on Central Pain Modulation in Chronic Whiplash and Fibromyalgia Patients Compared to Healthy Controls.* Pain Physician, 2016. **19**(3): p. 119-30.
2. Coppieters, I., et al., *Cognitive Performance Is Related to Central Sensitization and Health-related Quality of Life in Patients with Chronic Whiplash-Associated Disorders and Fibromyalgia.* Pain Physician, 2015. **18**(3): p. E389-401.
3. Borchers, A.T. and M.E. Gershwin, *Fibromyalgia: A Critical and Comprehensive Review.* Clin Rev Allergy Immunol, 2015. **49**(2): p. 100-51.
4. Mezhov, V., E. Guymer, and G. Littlejohn, *Central Sensitivity and Fibromyalgia.* Intern Med J, 2021.
5. Trouvin, A.P. and S. Perrot, *New concepts of pain.* Best Pract Res Clin Rheumatol, 2019. **33**(3): p. 101415.
6. Fitzcharles, M.A., et al., *Nociplastic pain: towards an understanding of prevalent pain conditions.* Lancet, 2021. **397**(10289): p. 2098-2110.
7. Blumenstiel, K., et al., *Quantitative sensory testing profiles in chronic back pain are distinct from those in fibromyalgia.* Clin J Pain, 2011. **27**(8): p. 682-90.
8. de Tommaso, M., E. Vecchio, and M. Nolano, *The puzzle of fibromyalgia between central sensitization syndrome and small fiber neuropathy: a narrative review on neurophysiological and morphological evidence.* Neurol Sci, 2022.
9. Harte, S.E., R.E. Harris, and D.J. Clauw, *The neurobiology of central sensitization.* Journal of Applied Biobehavioral Research, 2018. **23**(2): p. e12137.
10. den Boer, C., et al., *Central sensitization in chronic pain and medically unexplained symptom research: A systematic review of definitions, operationalizations and measurement instruments.* J Psychosom Res, 2019. **117**: p. 32-40.
11. Woolf, C.J., *Central sensitization: implications for the diagnosis and treatment of pain.* Pain, 2011. **152**(3 Suppl): p. S2-s15.
12. Oudejans, L., et al., *Cornea nerve fiber quantification and construction of phenotypes in patients with fibromyalgia.* Sci Rep, 2016. **6**: p. 23573.
13. Price, D.D., et al., *Enhanced temporal summation of second pain and its central modulation in fibromyalgia patients.* Pain, 2002. **99**(1-2): p. 49-59.
14. Staud, R., et al., *Cutaneous C-fiber pain abnormalities of fibromyalgia patients are specifically related to temporal summation.* Pain, 2008. **139**(2): p. 315-23.
15. Staud, R., et al., *Temporal summation of pain from mechanical stimulation of muscle tissue in normal controls and subjects with fibromyalgia syndrome.* Pain, 2003. **102**(1-2): p. 87-95.
16. Staud, R., et al., *Spatial summation of mechanically evoked muscle pain and painful aftersensations in normal subjects and fibromyalgia patients.* Pain, 2007. **130**(1-2): p. 177-87.
17. Staud, R., et al., *Abnormal sensitization and temporal summation of second pain (wind-up) in patients with fibromyalgia syndrome.* Pain, 2001. **91**(1-2): p. 165-175.
18. Staud, R., et al., *Spatial summation of heat pain within and across dermatomes in fibromyalgia patients and pain-free subjects.* Pain, 2004. **111**(3): p. 342-50.
19. Bourke, J.H., et al., *Central sensitisation in chronic fatigue syndrome and fibromyalgia; a case control study.* J Psychosom Res, 2021. **150**: p. 110624.
20. Goubert, D., et al., *Differences in Pain Processing Between Patients with Chronic Low Back Pain, Recurrent Low Back Pain, and Fibromyalgia.* Pain Physician, 2017. **20**(4): p. 307-318.
21. Gracely, R.H., M.A.B. Grant, and T. Giesecke, *Evoked pain measures in fibromyalgia.* Best Practice & Research Clinical Rheumatology, 2003. **17**(4): p. 593-609.
22. Treede, R.D., et al., *Pain elicited by blunt pressure: neurobiological basis and clinical relevance.* Pain, 2002. **98**(3): p. 235-240.
23. Arroyo-Fernandez, R., et al., *Pressure-Induced Referred Pain as a Biomarker of Pain Sensitivity in Fibromyalgia.* Pain Physician, 2020. **23**(4): p. E353-e362.
24. Harris, R.E., et al., *Elevated insular glutamate in fibromyalgia is associated with experimental pain.* Arthritis Rheum, 2009. **60**(10): p. 3146-52.
25. Witting, N., et al., *Intramuscular and intradermal injection of capsaicin: a comparison of local and referred pain.* PAIN, 2000. **84**(2): p. 407-412.

26. Middlebrook, N., et al., *Measures of central sensitization and their measurement properties in musculoskeletal trauma: A systematic review.* Eur J Pain, 2021. **25**(1): p. 71-87.
27. Henriksson, K.G., *Fibromyalgia--from syndrome to disease. Overview of pathogenetic mechanisms.* J Rehabil Med, 2003(41 Suppl): p. 89-94.
28. Littlejohn, G. and E. Guymer, *Modulation of NMDA Receptor Activity in Fibromyalgia.* Biomedicines, 2017. **5**(2).
29. Nijs, J., et al., *Treatment of central sensitization in patients with 'unexplained' chronic pain: what options do we have?* Expert Opin Pharmacother, 2011. **12**(7): p. 1087-98.
30. Arendt-Nielsen, L., et al., *The effect of N-methyl-D-aspartate antagonist (ketamine) on single and repeated nociceptive stimuli: a placebo-controlled experimental human study.* Anesth Analg, 1995. **81**(1): p. 63-8.
31. Graven-Nielsen, T., et al., *Ketamine reduces muscle pain, temporal summation, and referred pain in fibromyalgia patients.* Pain, 2000. **85**(3): p. 483-91.
32. Sörensen, J., et al., *Fibromyalgia--are there different mechanisms in the processing of pain? A double blind crossover comparison of analgesic drugs.* J Rheumatol, 1997. **24**(8): p. 1615-21.
33. Sorensen, J., et al., *Pain analysis in patients with fibromyalgia. Effects of intravenous morphine, lidocaine, and ketamine.* Scand J Rheumatol, 1995. **24**(6): p. 360-5.
34. Olivan-Blazquez, B., et al., *Efficacy of memantine in the treatment of fibromyalgia: A double-blind, randomised, controlled trial with 6-month follow-up.* Pain, 2014. **155**(12): p. 2517-2525.
35. Cohen, S.P., et al., *Consensus Guidelines on the Use of Intravenous Ketamine Infusions for Chronic Pain From the American Society of Regional Anesthesia and Pain Medicine, the American Academy of Pain Medicine, and the American Society of Anesthesiologists.* Reg Anesth Pain Med, 2018. **43**(5): p. 521-546.
36. Mangnus, T.J.P., et al., *Ketamine therapy for chronic pain in The Netherlands: a nationwide survey.* Scand J Pain, 2021.
37. Derry, S., et al., *Pregabalin for pain in fibromyalgia in adults.* Cochrane Database Syst Rev, 2016. **9**(9): p. Cd011790.
38. Derry, S., et al., *Milnacipran for neuropathic pain and fibromyalgia in adults.* Cochrane Database Syst Rev, 2012. **3**(3): p. Cd008244.
39. Arnold, L.M., et al., *Efficacy of duloxetine in patients with fibromyalgia: pooled analysis of 4 placebo-controlled clinical trials.* Prim Care Companion J Clin Psychiatry, 2009. **11**(5): p. 237-44.
40. Arnold, L.M., et al., *Gabapentin in the treatment of fibromyalgia: a randomized, double-blind, placebo-controlled, multicenter trial.* Arthritis & Rheumatism, 2007. **56**(4): p. 1336-1344.
41. Atzeni, F., et al., *An update on emerging drugs for fibromyalgia treatment.* Expert Opin Emerg Drugs, 2017. **22**(4): p. 357-367.
42. Macfarlane, G.J., et al., *EULAR revised recommendations for the management of fibromyalgia.* Ann Rheum Dis, 2017. **76**(2): p. 318-328.
43. Chen, X., et al., *The placebo effect and its determinants in fibromyalgia: meta-analysis of randomised controlled trials.* Clin Rheumatol, 2017. **36**(7): p. 1623-1630.
44. Zhang, W., et al., *The placebo effect and its determinants in osteoarthritis: meta-analysis of randomised controlled trials.* Ann Rheum Dis, 2008. **67**(12): p. 1716-23.
45. Evers, A.W.M., et al., *Implications of Placebo and Nocebo Effects for Clinical Practice: Expert Consensus.* Psychother Psychosom, 2018. **87**(4): p. 204-210.
46. Benedetti, F., *Placebo and the new physiology of the doctor-patient relationship.* Physiol Rev, 2013. **93**(3): p. 1207-46.
47. Colloca, L., *The Placebo Effect in Pain Therapies.* Annu Rev Pharmacol Toxicol, 2019. **59**: p. 191-211.
48. Colloca, L., P. Enck, and D. DeGrazia, *Relieving pain using dose-extending placebos: a scoping review.* Pain, 2016. **157**(8): p. 1590-8.
49. Doering, B.K. and W. Rief, *Utilizing placebo mechanisms for dose reduction in pharmacotherapy.* Trends Pharmacol Sci, 2012. **33**(3): p. 165-72.
50. Breivik, H., et al., *Assessment of pain.* Br J Anaesth, 2008. **101**(1): p. 17-24.

51. Dworkin, R.H., et al., *Core outcome measures for chronic pain clinical trials: IMMPACT recommendations.* PAIN, 2005. **113**(1): p. 9-19.
52. Cowen, R., et al., *Assessing pain objectively: the use of physiological markers.* Anaesthesia, 2015. **70**(7): p. 828-47.
53. Jafari, H., et al., *Pain and respiration: a systematic review.* Pain, 2017. **158**(6): p. 995-1006.
54. van den Bosch, O.F.C., et al., *Breathing variability-implications for anaesthesiology and intensive care.* Crit Care, 2021. **25**(1): p. 280.
55. Wysocki, M., et al., *Reduced breathing variability as a predictor of unsuccessful patient separation from mechanical ventilation.* Crit Care Med, 2006. **34**(8): p. 2076-83.
56. Wu, J.G., et al., *The effect of acute exposure to morphine on breathing variability and cardiopulmonary coupling in men with obstructive sleep apnea: A randomized controlled trial.* J Sleep Res, 2020. **29**(2): p. e12930.
57. Eikermann, M., et al., *Ketamine activates breathing and abolishes the coupling between loss of consciousness and upper airway dilator muscle dysfunction.* Anesthesiology, 2012. **116**(1): p. 35-46.
58. van den Bosch, O.F.C., et al., *Breathing variability during propofol/remifentanil procedural sedation with a single additional dose of midazolam or s-ketamine: a prospective observational study.* J Clin Monit Comput, 2021.
59. Benedetti, F., et al., *Conscious Expectation and Unconscious Conditioning in Analgesic, Motor, and Hormonal Placebo/Nocebo Responses.* The Journal of Neuroscience, 2003. **23**(10): p. 4315-4323.
60. Middlebrook, N., et al., *Reliability of temporal summation, thermal and pressure pain thresholds in a healthy cohort and musculoskeletal trauma population.* PLoS One, 2020. **15**(5): p. e0233521.
61. Graven-Nielsen, T. and L. Arendt-Nielsen, *Assessment of mechanisms in localized and widespread musculoskeletal pain.* Nat Rev Rheumatol, 2010. **6**(10): p. 599-606.
62. Au Yeung, S.T., et al., *Partial reinforcement, extinction, and placebo analgesia.* Pain, 2014. **155**(6): p. 1110-1117.
63. Bennett, R.M., et al., *The Revised Fibromyalgia Impact Questionnaire (FIQR): validation and psychometric properties.* Arthritis Res Ther, 2009. **11**(4): p. R120.
64. Noppers, I., et al., *Absence of long-term analgesic effect from a short-term S-ketamine infusion on fibromyalgia pain: a randomized, prospective, double blind, active placebo-controlled trial.* Eur J Pain, 2011. **15**(9): p. 942-9.
65. Robinson, B.R., et al., *Psychometric analysis of subjective sedation scales in critically ill adults.* Crit Care Med, 2013. **41**(9 Suppl 1): p. S16-29.
66. Zanos, P., et al., *Ketamine and Ketamine Metabolite Pharmacology: Insights into Therapeutic Mechanisms.* Pharmacol Rev, 2018. **70**(3): p. 621-660.
67. Niesters, M., C. Martini, and A. Dahan, *Ketamine for chronic pain: risks and benefits.* Br J Clin Pharmacol, 2014. **77**(2): p. 357-67.
68. Voscopoulos, C., et al., *Special article: evaluation of a novel noninvasive respiration monitor providing continuous measurement of minute ventilation in ambulatory subjects in a variety of clinical scenarios.* Anesth Analg, 2013. **117**(1): p. 91-100.
69. Benedetti, F., et al., *Nonopioid placebo analgesia is mediated by CB1 cannabinoid receptors.* Nat Med, 2011. **17**(10): p. 1228-30.
70. Lakens, D., *Calculating and reporting effect sizes to facilitate cumulative science: a practical primer for t-tests and ANOVAs.* Frontiers in Psychology, 2013. **4**.
71. Pourhoseingholi, M.A., M. Vahedi, and M. Rahimzadeh, *Sample size calculation in medical studies.* Gastroenterol Hepatol Bed Bench, 2013. **6**(1): p. 14-7.
72. Abdollahpour, A., E. Saffarieh, and B.H. Zoroufchi, *A review on the recent application of ketamine in management of anesthesia, pain, and health care.* J Family Med Prim Care, 2020. **9**(3): p. 1317-1324.
73. Peltoniemi, M.A., et al., *Ketamine: A Review of Clinical Pharmacokinetics and Pharmacodynamics in Anesthesia and Pain Therapy.* Clinical Pharmacokinetics, 2016. **55**(9): p. 1059-1077.

74. Sigtermans, M., et al., *An observational study on the effect of S+-ketamine on chronic pain versus experimental acute pain in Complex Regional Pain Syndrome type 1 patients.* Eur J Pain, 2010. **14**(3): p. 302-7.
75. Dahanl, A., et al., *Population pharmacokinetic-pharmacodynamic modeling of ketamine-induced pain relief of chronic pain.* 2011. **15**(3): p. 258-267.
76. Sigtermans, M.J., et al., *Ketamine produces effective and long-term pain relief in patients with Complex Regional Pain Syndrome Type 1.* PAIN, 2009. **145**(3): p. 304-311.
77. Prosenz, J. and B. Gustorff, *Midazolam as an active placebo in 3 fentanyl-validated nociceptive pain models.* PAIN, 2017. **158**(7).
78. Besson, M., et al., *GABAergic modulation in central sensitization in humans: a randomized placebo-controlled pharmacokinetic-pharmacodynamic study comparing clobazam with clonazepam in healthy volunteers.* Pain, 2015. **156**(3): p. 397-404.
79. Vuilleumier, P.H., et al., *Evaluation of anti-hyperalgesic and analgesic effects of two benzodiazepines in human experimental pain: a randomized placebo-controlled study.* PLoS One, 2013. **8**(3): p. e43896.
80. Kravitz, H.M., et al., *Alprazolam and Ibuprofen in the Treatment of Fibromyalgia-Report of a Double-Blind Placebo-Controlled Study.* Journal of Musculoskeletal Pain, 1994. **2**(1): p. 3-27.
81. Russell, I.J., et al., *Treatment of primary fibrositis/fibromyalgia syndrome with ibuprofen and alprazolam. A double-blind, placebo-controlled study.* Arthritis Rheum, 1991. **34**(5): p. 552-60.
82. Moldofsky, H., et al., *The effect of zolpidem in patients with fibromyalgia: a dose ranging, double blind, placebo controlled, modified crossover study.* (0315-162X (Print)).
83. Amanzio, M. and F. Benedetti, *Neuropharmacological dissection of placebo analgesia: expectation-activated opioid systems versus conditioning-activated specific subsystems.* J Neurosci, 1999. **19**(1): p. 484-94.
84. Niesters, M., et al., *Effect of ketamine on endogenous pain modulation in healthy volunteers.* Pain, 2011. **152**(3): p. 656-63.
85. Sin, B., et al., *The Use of Ketamine for Acute Treatment of Pain: A Randomized, Double-Blind, Placebo-Controlled Trial.* J Emerg Med, 2017. **52**(5): p. 601-608.
86. Niesters, M., et al., *Effect of Subanesthetic Ketamine on Intrinsic Functional Brain Connectivity.* Anesthesiology, 2012. **117**(4): p. 868-877.
87. Olkkola, K.T. and J. Ahonen, *Midazolam and other benzodiazepines.* Handb Exp Pharmacol, 2008(182): p. 335-60.
88. Severs, D., E.J. Hoorn, and M.B. Rookmaaker, *A critical appraisal of intravenous fluids: from the physiological basis to clinical evidence.* Nephrol Dial Transplant, 2015. **30**(2): p. 178-87.
89. Short, B., et al., *Side-effects associated with ketamine use in depression: a systematic review.* The Lancet Psychiatry, 2018. **5**(1): p. 65-78.
90. Katalinic, N., et al., *Ketamine as a new treatment for depression: A review of its efficacy and adverse effects.* Australian & New Zealand Journal of Psychiatry, 2013. **47**(8): p. 710-727.
91. Persson, J., et al., *The analgesic effect of racemic ketamine in patients with chronic ischemic pain due to lower extremity arteriosclerosis obliterans.* Acta anaesthesiologica scandinavica, 1998. **42**(7): p. 750-758.
92. Xu, J. and H. Lei, *Ketamine-an update on its clinical uses and abuses.* CNS Neurosci Ther, 2014. **20**(12): p. 1015-20.
93. Sinner, B. and B.M. Graf, *Ketamine*, in *Modern Anesthetics*, J. Schüttler and H. Schwilden, Editors. 2008, Springer Berlin Heidelberg: Berlin, Heidelberg. p. 313-333.
94. Trimmel, H., et al., *S(+)-ketamine : Current trends in emergency and intensive care medicine.* Wien Klin Wochenschr, 2018. **130**(9-10): p. 356-366.
95. Niesters, M., et al., *Influence of ketamine and morphine on descending pain modulation in chronic pain patients: a randomized placebo-controlled cross-over proof-of-concept study.* 2013. **110**(6): p. 1010-1016.
96. Sprenger, T., et al., *Imaging Pain Modulation by Subanesthetic S-(+)-Ketamine.* Anesthesia & Analgesia, 2006. **103**(3).

97. Koppert, W., et al., *A new model of electrically evoked pain and hyperalgesia in human skin: the effects of intravenous alfentanil, S(+)-ketamine, and lidocaine.* Anesthesiology, 2001. **95**(2): p. 395-402.
98. Koppert, W., et al., *Differential Modulation of Remifentanil-induced Analgesia and Postinfusion Hyperalgesia by S -Ketamine and Clonidine in Humans.* Anesthesiology, 2003. **99**: p. 152-159.
99. Kanto, J.H., *Midazolam: the first water-soluble benzodiazepine. Pharmacology, pharmacokinetics and efficacy in insomnia and anesthesia.* Pharmacotherapy, 1985. **5**(3): p. 138-55.
100. Weinbroum, A., et al., *The midazolam-induced paradox phenomenon is reversible by flumazenil. Epidemiology, patient characteristics and review of the literature.* European journal of anaesthesiology, 2001. **18**(12): p. 789-797.
101. Reves, J.G., M.D., et al., *Midazolam: Pharmacology and Uses.* Anesthesiology: The Journal of the American Society of Anesthesiologists, 1985. **62**(3): p. 310-324.
102. Barends, C.R., et al., *Dexmedetomidine versus Midazolam in Procedural Sedation. A Systematic Review of Efficacy and Safety.* PLoS One, 2017. **12**(1): p. e0169525.
103. Conway, A., J. Rolley, and J.R. Sutherland, *Midazolam for sedation before procedures.* Cochrane Database of Systematic Reviews, 2016(5).
104. Rodrigo, M.R. and J.B. Rosenquist, *Effect of conscious sedation with midazolam on oxygen saturation.* J Oral Maxillofac Surg, 1988. **46**(9): p. 746-50.
105. Coughlin, M.W. and H.J. Panuska, *Direct comparison of midazolam and diazepam for conscious sedation in outpatient oral surgery.* Anesth Prog, 1989. **36**(4-5): p. 160-3.
106. Biswas, S., et al., *Low-dose midazolam infusion for oculoplastic surgery under local anesthesia.* Eye (Lond), 1999. **13 (Pt 4)**: p. 537-40.
107. Murrough, J.W., et al., *Antidepressant efficacy of ketamine in treatment-resistant major depression: a two-site randomized controlled trial.* Am J Psychiatry, 2013. **170**(10): p. 1134-42.
108. Murrough, J.W., et al., *Ketamine for rapid reduction of suicidal ideation: a randomized controlled trial.* Psychological Medicine, 2015. **45**(16): p. 3571-3580.
109. Nordt, S.P. and R.F. Clark, *Midazolam: A review of therapeutic uses and toxicity.* The Journal of Emergency Medicine, 1997. **15**(3): p. 357-365.
110. Di Leo, E., et al., *Focus on the agents most frequently responsible for perioperative anaphylaxis.* Clin Mol Allergy, 2018. **16**: p. 16.
111. Barker, M.E., *0.9% saline induced hyperchloremic acidosis.* J Trauma Nurs, 2015. **22**(2): p. 111-6.
112. Brugnolli, A., et al., *Fluid Therapy Management in Hospitalized Patients: Results From a Cross-sectional Study.* Clin Ther, 2017. **39**(2): p. 311-321.
113. Bell, R.F. and E.A. Kalso, *Ketamine for pain management.* Pain Rep, 2018. **3**(5): p. e674.
114. Jin, J.F., et al., *The optimal choice of medication administration route regarding intravenous, intramuscular, and subcutaneous injection.* Patient Prefer Adherence, 2015. **9**: p. 923-42.
115. Haas, D.A. and D.G. Harper, *Ketamine: a review of its pharmacologic properties and use in ambulatory anesthesia.* Anesth Prog, 1992. **39**(3): p. 61-8.
116. Edwards, N.D., et al., *Combined infusions of morphine and ketamine for postoperative pain in elderly patients.* 2007. **48**(2): p. 124-127.
117. Hartvig, P., et al., *Central nervous system effects of subdissociative doses of (S)-ketamine are related to plasma and brain concentrations measured with positron emission tomography in healthy volunteers.* Clin Pharmacol Ther, 1995. **58**(2): p. 165-73.
118. Shafer, A., *Complications of sedation with midazolam in the intensive care unit and a comparison with other sedative regimens.* Critical Care Medicine, 1998. **26**(5).
119. Barr, J., et al., *A double-blind, randomized comparison of iv lorazepam versus midazolam for sedation of ICU patients via a pharmacologic model.* Anesthesiology: The Journal of the American Society of Anesthesiologists, 2001. **95**(2): p. 286-298.
120. Driessen, J.J., et al., *Continuous infusion of midazolam during anaesthesia and postoperative sedation after maxillofacial surgery.* Acta Anaesthesiologica Scandinavica, 1989. **33**(2): p. 116-121.

121. Klotz, U., et al., *Pharmacodynamic interaction between midazolam and a specific benzodiazepine antagonist in humans.* J Clin Pharmacol, 1985. **25**(6): p. 400-6.
122. Snidvongs, K. and S. Thanaviratananich, *Update on Intranasal Medications in Rhinosinusitis.* Curr Allergy Asthma Rep, 2017. **17**(7): p. 47.
123. Geisser, M.E., et al., *The association between experimental and clinical pain measures among persons with fibromyalgia and chronic fatigue syndrome.* Eur J Pain, 2007. **11**(2): p. 202-7.
124. Harris, R.E., et al., *Comparison of clinical and evoked pain measures in fibromyalgia.* J Pain, 2006. **7**(7): p. 521-7.
125. van Vliet, J., et al., *Qualitative and Quantitative Aspects of Pain in Patients With Myotonic Dystrophy Type 2.* The Journal of Pain, 2018. **19**(8): p. 920-930.
126. van de Donk, T., et al., *An experimental randomized study on the analgesic effects of pharmaceutical-grade cannabis in chronic pain patients with fibromyalgia.* Pain, 2019. **160**(4): p. 860-869.
127. Rolke, R., et al., *Quantitative sensory testing: a comprehensive protocol for clinical trials.* Eur J Pain, 2006. **10**(1): p. 77-88.
128. Tunks, E., et al., *Tender points in fibromyalgia.* Pain, 1988. **34**(1): p. 11-9.
129. Graven-Nielsen, T., et al., *Assessment of musculoskeletal pain sensitivity and temporal summation by cuff pressure algometry: a reliability study.* PAIN, 2015. **156**(11).
130. Price, D.D. and R. Dubner, *Mechanisms of first and second pain in the peripheral and central nervous systems.* J Invest Dermatol, 1977. **69**(1): p. 167-71.
131. Woolf, C.J. and M.W. Salter, *Neuronal Plasticity: Increasing the Gain in Pain.* Science, 2000. **288**(5472): p. 1765-1768.
132. Williams, D.A. and L.M. Arnold, *Measures of fibromyalgia: Fibromyalgia Impact Questionnaire (FIQ), Brief Pain Inventory (BPI), Multidimensional Fatigue Inventory (MFI-20), Medical Outcomes Study (MOS) Sleep Scale, and Multiple Ability Self-Report Questionnaire (MASQ).* Arthritis Care Res (Hoboken), 2011. **63 Suppl 11**: p. S86-97.
133. Bowdle, T.A., et al., *Psychedelic effects of ketamine in healthy volunteers: relationship to steady-state plasma concentrations.* Anesthesiology, 1998. **88**(1): p. 82-8.
134. Jonkman, K., et al., *Differential role of nitric oxide in the psychedelic symptoms induced by racemic ketamine and esketamine in human volunteers.* British Journal of Anaesthesia, 2018. **120**(5): p. 1009-1018.
135. Pallant, J.F. and C.M. Bailey, *Assessment of the structure of the Hospital Anxiety and Depression Scale in musculoskeletal patients.* Health Qual Life Outcomes, 2005. **3**: p. 82.
136. LoMartire, R., et al., *Psychometric properties of Short Form-36 Health Survey, EuroQol 5-dimensions, and Hospital Anxiety and Depression Scale in patients with chronic pain.* Pain, 2020. **161**(1): p. 83-95.
137. Spinhoven, P., et al., *A validation study of the Hospital Anxiety and Depression Scale (HADS) in different groups of Dutch subjects.* Psychol Med, 1997. **27**(2): p. 363-70.
138. O'Brien, A.T., et al., *Defective Endogenous Pain Modulation in Fibromyalgia: A Meta-Analysis of Temporal Summation and Conditioned Pain Modulation Paradigms.* The Journal of Pain, 2018. **19**(8): p. 819-836.
139. Raz, N., Y. Granovsky, and R. Defrin, *Investigating the neural processing of spatial summation of pain: the role of A-delta nociceptors.* Exp Brain Res, 2015. **233**(2): p. 405-13.
140. van Dorp, E., et al., *Naloxone reversal of buprenorphine-induced respiratory depression.* Anesthesiology, 2006. **105**(1): p. 51-7.
141. Goldfrank, L., et al., *A dosing nomogram for continuous infusion intravenous naloxone.* Annals of Emergency Medicine, 1986. **15**(5): p. 566-570.
142. Team, R.S., *RStudio: Integrated Development for R*. 2020, RStudio PBC: Boston.
143. Watkins, M.P. and L. Portney, *Foundations of clinical research: applications to practice*. 2009: Pearson/Prentice Hall Upper Saddle River, NJ.
144. Hox, J. and M. Moerbeek, *Multilevel analysis: Techniques and applications. Routledge.* New York, 2010.

145. Hayes, A.F. and N.J. Rockwood, *Regression-based statistical mediation and moderation analysis in clinical research: Observations, recommendations, and implementation.* Behaviour Research and Therapy, 2017. **98**: p. 39-57.
146. Guedj, E., et al., *Follow-up of pain processing recovery after ketamine in hyperalgesic fibromyalgia patients using brain perfusion ECD-SPECT.* 2007. **34**(12): p. 2115-2119.
147. Hermans, L., et al., *Influence of Morphine and Naloxone on Pain Modulation in Rheumatoid Arthritis, Chronic Fatigue Syndrome/Fibromyalgia, and Controls: A Double-Blind, Randomized, Placebo-Controlled, Cross-Over Study.* Pain Pract, 2018. **18**(4): p. 418-430.
148. Amrein, R., et al., *Clinical pharmacology of Dormicum (midazolam) and Anexate (flumazenil).* Resuscitation, 1988. **16**: p. S5-S27.
149. Bosma, R.L., et al., *Brain Dynamics and Temporal Summation of Pain Predicts Neuropathic Pain Relief from Ketamine Infusion.* Anesthesiology, 2018. **129**(5): p. 1015-1024.
150. Orhurhu, V., et al., *Ketamine Infusions for Chronic Pain: A Systematic Review and Meta-analysis of Randomized Controlled Trials.* Anesth Analg, 2019. **129**(1): p. 241-254.
151. Takeda, Y., et al., *Patient-oriented optimal depth of conscious sedation using midazolam during flexible bronchoscopy: A prospective open-labeled single-arm trial.* Respiratory Investigation, 2018. **56**(4): p. 349-355.
