# Supplementary material for: Effects of s-ketamine and midazolam on respiratory variability: A randomized controlled pilot trial
Source: PLoS One. 2025 Sep 4;20(9):e0331358. doi: 10.1371/journal.pone.0331358 (PMC12410786; doi:10.1371/journal.pone.0331358)
Supplement: Attachment — Submitted filename: Journal review of Midazolam Vs S-ketamine.docx [file pone.0331358.s004.docx]

Journal review of “*Effects of S-Ketamine and Midazolam on Respiratory Variability: A Randomized Controlled Pilot Trial”*

# 1. Brief Summary

This study investigates midazolam's respiratory depressant effects compared to S-ketamine. The authors use a noninvasive bioimpedance method to assess respiratory rate and tidal volume variations (VRR and VTV). Their findings show that midazolam does not significantly decrease respiratory rate but reduces VRR, impairing respiratory system adaptability. This decrease in respiratory rate and tidal volume resembles differences between successfully extubated patients and those who failed extubation trials.

There are notable limitations to this study, and the authors may have overextended their conclusions. The study originated from a trial examining the anesthetic effects of S-ketamine and midazolam in fibromyalgia patients without any respiratory system abnormality, but the dosing regimen or the drug effect may differ when used for conscious sedation or intubated ICU patients with significant respiratory comorbidities. Only 78% of the respiratory data were available, which could significantly impact the ultimate result. Additionally, while the noninvasive bioimpedance technique for measuring tidal volume shows potential, broader acceptance and implementation in clinical practice are still needed.

# 2. Comments to the authors

I commend the authors for conducting a well-protocolized study on the respiratory depression effects of midazolam compared to S-ketamine. The findings suggest that S-ketamine may be a safer option for protecting the respiratory system's adaptability.

Although suppression of respiratory drive is a well-recognized effect of benzodiazepines via GABA-ergic stimulation, addressing a few remaining questions would enhance the study's clarity and impact. Some revisions could help present the findings more accurately and strengthen the overall conclusions.

## A. The noninvasive bioimpedance technique (ExSpiron®Xi) has shown validation in some smaller trials compared to spirometry. However, the respiratory rate (VRR) and tidal volume (VTV) derived from bioimpedance need wider acceptance in clinical practice. While the bioimpedance-derived respiratory rate and tidal volume correlate well with spirometry, minute ventilation does not. This study focuses on non-invasive monitoring of respiratory variability, and we are uncertain if these findings are reproducible in similar studies or in clinical practice.

## B. Respiratory data were only available in 78% of the visits. The remaining 22% missing data could have significantly altered the study’s findings.

## C. In the study, the maximum dosing was 0.3 mg/kg/h for S-ketamine and 0.05 mg/kg/h for midazolam. The authors did not provide participants' median body weight, but the median BMI was 27.7. For an 80 kg participant, this translates to a maximum dose of 24 mg/h of S-ketamine and 4 mg/h of midazolam. The dosing regimen in clinical practice may significantly differ, potentially affecting the respiratory depressive effects of these medications.

## D. In the discussion section (page 18, line 222), the author states, “Interestingly, the magnitude of this decrease in respiratory rate and tidal volume is similar to the differences seen in the intensive care unit between patients who fail and succeed at extubating attempts following prolonged ventilation.” However, I believe this interpretation stretches the study's findings. The research focused on fibromyalgia patients who were otherwise healthy and not on other sedatives/analgesics. In the ICU, many intubated patients have respiratory issues and may be receiving fentanyl or other analgesics along with sedatives. Thus, the VRR or VTV observed here might not apply to ICU patients.
