# Supplementary material for: Effects of s-ketamine and midazolam on respiratory variability: A randomized controlled pilot trial
Source: PLoS One. 2025 Sep 4;20(9):e0331358. doi: 10.1371/journal.pone.0331358 (PMC12410786; doi:10.1371/journal.pone.0331358)
Supplement: Attachment — Submitted filename: Response to the reviewers.docx [file pone.0331358.s006.docx]

**Response to Reviewer #1**

The authors provide an appropriate background that clearly introduces the concept of respiratory variability, its clinical importance, and the current knowledge gaps in the field. This context is helpful in establishing the relevance and need for the study. The manuscript is appropriately referenced, citing key studies that support the rationale for the investigation.

Thank you very much for your help with improving our manuscript.

However, a few areas warrant clarification and improvement:

Exclusion Criteria:

The exclusion criteria do not mention respiratory diseases, which is a significant omission. Given that such conditions can independently affect respiratory variability, it would be important to state whether participants with respiratory disorders were excluded.

Yes, we agree, respiratory diseases were part of the exclusion criteria. We have added this to the Methods section.

Terminology Consistency:

In line 89, the term “breathing variability” is used, while “respiratory variability” is used elsewhere. For consistency and clarity, the manuscript should consistently use “respiratory variability.”

Yes, we agree. We have corrected this throughout the manuscript.

Methods – Procedures Section:

The description of procedures lacks clarity and sufficient detail. Since the study specifically evaluates respiratory variability in response to ketamine and midazolam infusions, the inclusion of the 4th visit for saline only administration details appears unnecessary and may confuse readers. Consider streamlining this section by removing unrelated procedural details.

Yes, we agree. We have removed the 4^th^ visit from the Procedures section.

Dose Rationale:

The rationale behind the chosen doses and duration of ketamine and midazolam administration should be clearly explained. Are these doses based on an established protocol for fibromyalgia treatment?

Yes, we agree that this should be explained. The dose of ketamine was based on existing fibromyalgia studies and treatment protocols. The dose of midazolam was based on the goal to induce a mild sedative state. We added this to the Methods section with appropriate references.

Outcomes Section:

Lines 150–154, which describe the timing of respiratory recordings, would be more appropriately placed under the “Procedures” subsection. Additionally, clarification is needed regarding the decision to obtain measurements only during a 20-minute window starting 45 minutes after infusion initiation. Why were measurements not collected throughout the infusion period?

We agree that clarification is warranted. The study drugs were administered using a stepwise (step-up) infusion protocol, with increasing doses every 20 minutes. Our aim was to assess the physiological effects at the highest dose level, which began at t+40 minutes. Given the pharmacokinetic properties of the study drugs, particularly their rapid onset and short distribution half-life, we initiated the measurement interval at t+45 minutes to ensure that a steady-state concentration had been reached. The 20-minute measurement duration was chosen for pragmatic reasons, aligning with the 20-minute infusion period for each dosing step. This explanation has now been added to the Methods section.

Statistical Methods:

It appears that a quantitative time-series evaluation was used to assess respiratory variability. If so, this method should be explicitly stated under the “Statistical Analysis” section.

Yes, we agree, this is now added.

Missing Data:

In line 178, the manuscript notes that respiratory data were only available for 78% of visits. The authors should explain the reasons for the 22% data loss, especially since the study was conducted in a controlled environment.

Yes, we agree, and we have added the reasons for these missing data to the manuscript. Data were incomplete due to (i) missing baseline measurements, (ii) inadequate capture of respiratory signals, and (iii) inadequate storage of the recordings in the respiratory monitor.

Baseline Characteristics:

There are notable differences in baseline characteristics—e.g., median age (48 vs. 53) and weight (74 vs. 80)—between the ketamine and midazolam groups. Was a statistical comparison performed to determine whether these differences were significant and if they may have influenced the results?

We did not perform statistical comparisons of baseline characteristics between groups, as participants were allocated through randomization. Any observed differences at baseline are expected to occur by chance, and thus there is no underlying hypothesis to test. Conducting statistical tests in this context could lead to misinterpretation, as any statistically significant differences would, by definition, represent Type I errors. We think that statistical testing of baseline variables is not warranted. Although the mean was marginally different, the standard deviation/interquartile range overlap in both groups. Additionally, there are no pharmacokinetic differences to be expected with this marginal difference in weight or age

Table Content:

The table includes fibromyalgia impact scores. It would be helpful to clarify the relevance of this score to the study’s outcomes. Additionally, data from the saline group are presented in the table, but no corresponding respiratory variability data or analysis is provided or discussed. If this data is not directly relevant, consider removing it to maintain focus.

We agree that fibromyalgia impact scores are not directly relevant and have removed them from our manuscript. Data from the saline group were used in the linear mixed model to estimate effect sizes associated with s-ketamine or midazolam in comparison to saline.

Discussion Clarity:

In lines 222–225, the authors compare their findings to differences observed in ICU patients undergoing extubation trials. However, spontaneous breathing trials are typically performed when sedatives are withheld. The relevance of this comparison to the current study—where sedatives were actively administered—should be clarified and more appropriately discussed.

We agree and we have changed this section accordingly.

Overall, the manuscript explores an important and novel area, but addressing the points above will enhance its clarity, scientific rigor, and clinical applicability.

**Response to Reviewer #2**

Overall, well written manuscript. I would recommend adding a few more details (as below) if seems appropriate for the audience to better understand the study design.

Thank you very much for your help with improving our manuscript.

1). Did the saline group experience increase in rate of their saline infusion to better align with the increase in dosing for both Midazolam and S-ketamine (to minimize bias)
Yes, and we have added this to the Methods section of the manuscript.

2) Please add a clarification sentence that the same group of patients received the same medications (S-ketamine, midazolam or saline) all 3 times.

We agree that this is warranted and have made appropriate changes to the Methods section.

**Response to Reviewer #3**

I commend the authors for conducting a well-protocolized study on the respiratory depression effects of midazolam compared to S-ketamine. The findings suggest that S-ketamine may be a safer option for protecting the respiratory system's adaptability.

Although suppression of respiratory drive is a well-recognized effect of benzodiazepines via GABA-ergic stimulation, addressing a few remaining questions would enhance the study's clarity and impact. Some revisions could help present the findings more accurately and strengthen the overall conclusions.

Thank you very much for your help with improving our manuscript.

A. The noninvasive bioimpedance technique (ExSpiron®Xi) has shown validation in some smaller trials compared to spirometry. However, the respiratory rate (VRR) and tidal volume (VTV) derived from bioimpedance need wider acceptance in clinical practice. While the bioimpedance-derived respiratory rate and tidal volume correlate well with spirometry, minute ventilation does not. This study focuses on non-invasive monitoring of respiratory variability, and we are uncertain if these findings are reproducible in similar studies or in clinical practice.

We agree that the correlation between minute ventilation measured by bioimpedance-based techniques and spirometry is generally weaker compared to the correlation for respiratory rate and tidal volume. For this reason, we limited our analysis to respiratory rate and tidal volume, which are more reliably captured by the bioimpedance method. We also acknowledge the limited number of studies and the current lack of established clinical practices regarding non-invasive monitoring of respiratory variability. This is added to the Discussion.

B. Respiratory data were only available in 78% of the visits. The remaining 22% missing data could have significantly altered the study’s findings.

We agree that 22% of respiratory measurements were incomplete, and we have now added the specific reasons for this in the Results section. Given the technical nature of the missing data, we consider it most likely to be either Missing Completely At Random (MCAR) or Missing At Random (MAR). Therefore, we believe that the use of a linear mixed model, capable of handling such missingness, provides unbiased effect estimates and represents an appropriate analytical approach. We have reconfirmed this interpretation in consultation with our statistician. Additionally, we have acknowledged this as a limitation in the Discussion section.

C. In the study, the maximum dosing was 0.3 mg/kg/h for S-ketamine and 0.05 mg/kg/h for midazolam. The authors did not provide participants' median body weight, but the median BMI was 27.7. For an 80 kg participant, this translates to a maximum dose of 24 mg/h of S-ketamine and 4 mg/h of midazolam. The dosing regimen in clinical practice may significantly differ, potentially affecting the respiratory depressive effects of these medications.

Dosing strategies were based on existing fibromyalgia treatment protocols for s-ketamine, and for the goal to induce a conscious sedative state for midazolam. We agree that dosing regimens in clinical practice may differ, and we have added this to the limitations of the study. Of note, in a previous study, even smaller doses of s-ketamine and midazolam induced interesting changes in respiratory variability (Ref. 5). The participants’ weight is shown in Table 1.

D. In the discussion section (page 18, line 222), the author states, “Interestingly, the magnitude of this decrease in respiratory rate and tidal volume is similar to the differences seen in the intensive care unit between patients who fail and succeed at extubating attempts following prolonged ventilation.” However, I believe this interpretation stretches the study's findings. The research focused on fibromyalgia patients who were otherwise healthy and not on other sedatives/analgesics. In the ICU, many intubated patients have respiratory issues and may be receiving fentanyl or other analgesics along with sedatives. Thus, the VRR or VTV observed here might not apply to ICU patients.

We agree that more cautious interpretation is warranted and we have clarified this accordingly.

**Response to Reviewer #4**

This article presents results from a pilot study comparing ketamine to midazolam in terms of respiratory effects.

Thank you very much for your help with improving our manuscript.

There are several points which need clarification.

1. The background seems very brief and it is difficult to understand exactly what is novel about the work to be undertaken.

We appreciate the reviewer’s feedback and we were intending to provide a concise overview of the study aim. We are open to expanding this section but are unsure which aspects require elaboration.

2. Did each subject get each treatment? Or could a subject get the same treatment at each visit? Is this a crossover study? Or - each subject got the same treatment 3 times? Please describe more clearly. (I understand now they got the same treatment 3 times, but this is not clear as written.)

Yes, we agree and we have clarified this accordingly in the Methods section.

3. No sample size calculation is presented, but it is presented in the protocol. Please present a sample size calculation here.

We have added information about the sample size calculation to the Statistical Analysis section. As this manuscript focuses on respiratory variability, we believe that presenting the full sample size calculation, originally based on the primary aim of investigating pharmacological conditioning, is not directly relevant. However, we have clarified that the study was powered for the primary aim, as outlined in the original study protocol.

4. What about this study makes it a pilot? Please justify using this descriptor.

We labeled this study a pilot because it was embedded within a larger randomized controlled trial, and it was not powered for definitive hypothesis testing of this secondary aim. The pilot designation reflects both the limited sample size and the novel application of this respiratory monitoring approach in this context. We have clarified this in the Trial design section.

5. How was missing data dealt with?

We have added the reasons for missing data. Given the technical nature of the missing data, we consider it most likely to be either Missing Completely At Random (MCAR) or Missing At Random (MAR). Therefore, we believe that the use of a linear mixed model, capable of handling such missingness, provides unbiased effect estimates and represents an appropriate analytical approach. We have reconfirmed this interpretation in consultation with our statistician. Additionally, we have acknowledged this as a limitation in the Discussion section.

6. Were any subgroup analyses planned? Conducted?

Due to the small sample size and pilot nature of this study, there were no planned or conducted subgroup analyses.

7. The objective as written in the background only mentions respiratory variability, while rate, variability and variability of tidal volume are used as outcomes. In the outcomes it is described as "respiratory effects".

Yes, we have added this to the Background section.

8. Please indicate in the figure 2 whether ketamine is different from medazolam.

In the original manuscript, we did not test for significant differences between s-ketamine and midazolam. In response to this comment, we conducted a post hoc analysis using midazolam as the reference condition (instead of saline). This analysis revealed significant differences between s-ketamine and midazolam for all three respiratory parameters. We have updated the Methods, Results, Supplementary Information, and Figure 2 accordingly.

9. Figure 2 - did these results account for the repeated measures nature of the data? If not, please revise.

Yes, the results are based on the mixed effects model accounting for repeated measures.

10. Please give a more thoughtful consideration of missing data. Were the participants who dropped out different from those who completed all visits? How?

We agree that this information is warranted. Participants with incomplete data had a higher weight compared to those with complete data (84 [77 to 93] vs 73 [63 to 84] kg, p = 0.025), while other participant characteristics showed no significant differences. We have added this to the Results section.

11. Lines 229 in the discussion - please present these results in the results section as well. (See comment #8)

In the original manuscript, we did not test for significant differences between s-ketamine and midazolam. In response to this comment, we conducted a post hoc analysis using midazolam as the reference condition (instead of saline). This analysis revealed significant differences between s-ketamine and midazolam for all three respiratory parameters. We have updated the Methods, Results, Supplementary Information, and Figure 2 accordingly.

12. line 254 - unclear from results presented if the effects are different.

In the original manuscript, we did not test for significant differences between s-ketamine and midazolam. In response to this comment, we conducted a post hoc analysis using midazolam as the reference condition (instead of saline). This analysis revealed significant differences between s-ketamine and midazolam for all three respiratory parameters. We have updated the Methods, Results, Supplementary Information, and Figure 2 accordingly.

13. Please also mention the small sample size and high rate of missingness as limitations - what effect may this have on results?

We agree that this required clarification. We have added appropriate comments to the limitations section.

**Response to Reviewer #5**

Extremely good study signifying the preference for Ketamine to prevent respiratory depression.

Well know use of ketamine for conscious sedation for emergency procedures is justified with objective lung parameters that clearly shows that its a better choice in respiratory patients.

Preference of ketamine is justified at molecular level.

Thank you for taking the time to review our manuscript.

**Response to Reviewer #6**

This study has only 28 female patients, and needs a larger sample size.

We have added information that the sample size was estimated based on the primary aim of the randomized controlled trial, which was to assess pharmacological conditioning. Our pilot study can be used to determine appropriate sample sizes in future studies.

Following Corrections/ additions are my suggestions: S-ketamine is not used commonly in anesthesia, and Midazolam is also used as an anxiolytic.

Exclusion criteria: should include patients with COPD, severe OSA, on home oxygen, heavy smokers, and allergy to benzodiazepines and ketamine.

We have added this to the Methods section.

They need to add details of questionnaires.

We have removed the fibromyalgia impact score questionnaires and hospital anxiety and depression scale questionnaires from the manuscript to maintain clarity.

They should have monitored Co2, minute volume and VBGs.

We agree that measurements such as end-tidal CO₂, minute ventilation, and venous blood gases would have provided additional details on respiratory function. However, in this study, we intentionally focused on non-invasive measures of respiratory variability as the primary outcome. Future studies could build on this work by exploring correlations with more comprehensive respiratory parameters.

Paragraph one in Page 5 and 6 and outcomes section need better flow of English.

We have clarified these paragraphs.

Why did they choose this specific ketamine and Midazolam infusion dose?

Dosing strategies were based on existing fibromyalgia treatment protocols for s-ketamine, and for the goal to induce a mildly sedative state for midazolam. We have added this to the Methods section, with appropriate references.

Any info about quality and side-effects of sedation medications?

There were no notable side-effects or problems with quality of sedation medications.
